# Supplementary material for: COVID-19 and children with congenital anomalies: a European survey of parents’ experiences of healthcare services
Source: BMJ Open. 2022 Jul 19;12(7):e061428. doi: 10.1136/bmjopen-2022-061428 (PMC9301497; doi:10.1136/bmjopen-2022-061428)
Supplement: Supplementary data [file bmjopen-2022-061428supp001.pdf]

SUPPLEMENTARY FILE

(A) SURVEY ITEMS

(1) Parent Demographics

1. Which country do you live in? [drop-down list]

2. What type of area do you live in?
   a. City (population over 500,000)
   b. Large town (population between 100,000 and 500,000)
   c. Medium town (population between 20,000 and 100,000)
   d. Small town (population less than 20,000)
   e. Suburban village
   f. Village
   g. Rural/isolated area (e.g. a farm)

3. What is your age?
   a. Less than 20 years
   b. 20-25 years
   c. 26-30 years
   d. 31-35 years
   e. 36-40 years
   f. 41-45 years
   g. 46-50 years
   h. More than 50 years

4. What is the highest level of education you have completed?
   a. Primary school
   b. Secondary school up to 16 years
   c. Secondary or further education after 16 years
   d. University
   e. Post-graduate / Doctoral studies

5. What is your employment status?
   a. Employed (full-time), including self-employed
   b. Employed (part-time), including self-employed
   c. Full-time homemaker/carer
   d. Long-term sick/disabled
   e. Retired
   f. Student
   g. Unemployed
   h. On furlough

6. How long have you lived in your country of residence?
   a. Up to 1 year
   b. Between 1-5 years
   c. Between 6-10 years
   d. More than 10 years
   e. From birth

f. Prefer not to say

7. What is your relationship to the child this survey is about?

a. Mother (biological)
b. Mother (adoptive)
c. Father (biological)
d. Father (adoptive)
e. Legal guardian related to the child
f. Legal guardian unrelated to the child / foster parent
g. Another family member

8. Is your child being raised with any siblings?

a. Yes, biological sibling(s)
b. Yes, adoptive sibling(s)
c. Yes, biological and adoptive siblings
d. No [survey skips to next section]

9. Is their sibling/are their siblings older or younger than the child this survey is about?

a. Older
b. Younger
c. Both older and younger
d. Same age (twin)

(2) Child Demographics and Medical Information

1. What age is your child?

a. Less than 1 year
b. 1-3 years
c. 4-6 years
d. 7-10 years

2. What is your child's gender?

a. Male
b. Female
c. Other
d. Prefer not to say

3. Which of the following conditions has your child been diagnosed with? (If your child has <u>more than one</u> of these conditions, please select all that apply)

a. Cleft lip (with or without cleft palate)
b. Spina bifida
c. Congenital heart defect that required surgical intervention
d. Down's syndrome

4. Was your child's [*condition*] detected prenatally (during pregnancy)?

a. Yes [survey moves to question 5]
b. No [survey skips to question 6]
c. I don't know [survey skips to question 6]

5. In which week of pregnancy was your child's [*condition*] detected?

   a. Before 13 weeks
   b. Between 14 and 21 weeks
   c. At 22 weeks or later
   d. I'm not sure

6. Does your child have any other congenital anomalies (conditions present from birth)?

   a. Yes

   Please select all that apply:
   - Brain anomalies
   - Hydrocephalus
   - Eye anomalies
   - Anomalies of face, ear and neck
   - Lung anomalies
   - Abdominal anomalies
   - Renal anomalies
   - Genital anomalies
   - Skeletal anomalies
   - Limb anomalies
   - Chromosomal or genetic abnormality (other than Down's syndrome)
   - Other anomaly

   b. No

7. Does your child have any other health conditions?

   a. Yes

   Please select all that apply:
   - Autism or attention disorder
   - Learning disability
   - Epilepsy
   - Cerebral Palsy
   - Asthma
   - Allergy or food intolerance
   - Eczema or other skin disease
   - Recurrent infections
   - Hearing loss
   - Vision problems
   - Celiac disease
   - Diabetes
   - Endocrine disorder
   - Immune disorder
   - Blood disorder
   - Cancer
   - Other

   b. No

(3) Provision of healthcare

1. Has your child had any routine appointments postponed or cancelled?
   a. Yes
   b. No
   c. Not applicable (they have not had any routine appointments in this period)

2. Has your child had any planned surgeries postponed or cancelled?
   a. Yes
   b. No
   c. Not applicable (they have not had any planned surgeries in this period)

3. Has your child had any planned tests or procedure's postponed or cancelled?
   a. Yes
   b. No
   c. Not applicable (they have not had any planned tests or procedures in this period)

4. Has your child had any face-to-face appointments re-scheduled as virtual appointments (e.g. by telephone or online)?
   a. Yes
   b. No
   c. Not applicable (they have not had any appointments in this period)

5. [If yes] Overall, how do you rate the quality of your virtual appointments?
   a. Poor
   b. Fair
   c. Good
   d. Very good

7. Have you had any difficulty accessing medication for your child?
   a. Not at all
   b. A little
   c. Quite a bit
   d. Very much
   e. Not applicable (they do not require medication)

(4) Impact on the child

1. Has your child's health been compromised by any changes to ongoing treatment for their condition (e.g. medication, physical therapy, or surgery)?
   a. Not at all
   b. Slightly
   c. Moderately
   d. Severely
   e. Not applicable (there have been no changes to their ongoing treatment)
   f. Not applicable (they have not had any ongoing treatment)

Latos-Biele G6¶ ,A*et al. BMJ Open* 2022; 12:e061428. doi: 10.1136/bmjopen-2022-061428

2. Compared to before COVID-19, how would you rate your child's physical health?

a. Much worse
b. Somewhat worse
c. About the same
d. Somewhat better
e. Much better

3. Compared to before COVID-19, how would you rate your child's emotional well-being?

a. Much worse
b. Somewhat worse
c. About the same
d. Somewhat better
e. Much better

(5) Support for Parents

1. During the pandemic, to what extent have you felt satisfied with the support you have received from the following people/organisations? (If you have not needed or requested support from a listed source please select N/A)

	Not at all satisfied	Slightly satisfied	Moderately satisfied	Very satisfied	N/A
General practitioner					
Specialist doctor or specialist nurse					
Partner (or person I am closest to)					
Friends and family					
Parents of children with the same health condition					
Patient/parent organisation					
School					

2. Overall, would you have liked more support during the COVID-19 pandemic?

a. Not at all
b. A little
c. Quite a bit
d. Very much

(B) STROBE CHECKLIST

STROBE Statement—Checklist of items that should be included in reports of ***cross-sectional studies***

	Item No	Recommendation	Page No
**Title and abstract**	1	(*a*) Indicate the study's design with a commonly used term in the title or the abstract	1, 3
		(*b*) Provide in the abstract an informative and balanced summary of what was done and what was found	3
**Introduction**			
Background/rationale	2	Explain the scientific background and rationale for the investigation being reported	4-5
Objectives	3	State specific objectives, including any prespecified hypotheses	5
**Methods**			
Study design	4	Present key elements of study design early in the paper	5, 7
Setting	5	Describe the setting, locations, and relevant dates, including periods of recruitment, exposure, follow-up, and data collection	5-7
Participants	6	(*a*) Give the eligibility criteria, and the sources and methods of selection of participants	7
Variables	7	Clearly define all outcomes, exposures, predictors, potential confounders, and effect modifiers. Give diagnostic criteria, if applicable	7-8
Data sources/ measurement	8*	For each variable of interest, give sources of data and details of methods of assessment (measurement). Describe comparability of assessment methods if there is more than one group	7
Bias	9	Describe any efforts to address potential sources of bias	n/a
Study size	10	Explain how the study size was arrived at	9
Quantitative variables	11	Explain how quantitative variables were handled in the analyses. If applicable, describe which groupings were chosen and why	9
Statistical methods	12	(*a*) Describe all statistical methods, including those used to control for confounding	9-10
		(*b*) Describe any methods used to examine subgroups and interactions	9-10
		(*c*) Explain how missing data were addressed	9-10
		(*d*) If applicable, describe analytical methods taking account of sampling strategy	n/a
		(*e*) Describe any sensitivity analyses	n/a

Latos-Biele G6¶ ,Aet al. BMJ Open 2022; 12:e061428. doi: 10.1136/bmjopen-2022-061428

**Results**			
Participants	13*	(a) Report numbers of individuals at each stage of study—eg numbers potentially eligible, examined for eligibility, confirmed eligible, included in the study, completing follow-up, and analysed	10
		(b) Give reasons for non-participation at each stage	n/a
		(c) Consider use of a flow diagram	n/a
Descriptive data	14*	(a) Give characteristics of study participants (eg demographic, clinical, social) and information on exposures and potential confounders	6, 10
		(b) Indicate number of participants with missing data for each variable of interest	10 (and Tables 2-5)
Outcome data	15*	Report numbers of outcome events or summary measures	n/a
Main results	16	(*a*) Give unadjusted estimates and, if applicable, confounder-adjusted estimates and their precision (eg, 95% confidence interval). Make clear which confounders were adjusted for and why they were included	11-14, 16 (Tables 2-5)
		(*b*) Report category boundaries when continuous variables were categorized	n/a
		(*c*) If relevant, consider translating estimates of relative risk into absolute risk for a meaningful time period	n/a
Other analyses	17	Report other analyses done—eg analyses of subgroups and interactions, and sensitivity analyses	n/a
**Discussion**			
Key results	18	Summarise key results with reference to study objectives	17
Limitations	19	Discuss limitations of the study, taking into account sources of potential bias or imprecision. Discuss both direction and magnitude of any potential bias	18-19
Interpretation	20	Give a cautious overall interpretation of results considering objectives, limitations, multiplicity of analyses, results from similar studies, and other relevant evidence	17-18
Generalisability	21	Discuss the generalisability (external validity) of the study results	18
**Other information**			
Funding	22	Give the source of funding and the role of the funders for the present study and, if applicable, for the original study on which the present article is based	21-22

*Give information separately for exposed and unexposed groups. **Note:** An Explanation and Elaboration article discusses each checklist item and gives methodological background and published examples of transparent reporting. The STROBE checklist is best used in conjunction with this article (freely available on the Web sites of PLoS Medicine at http://www.plosmedicine.org/, Annals of Internal Medicine at http://www.annals.org/, and Epidemiology at http://www.epidem.com/). Information on the STROBE Initiative is available at www.strobe-statement.org.

(C) LOGISTIC REGRESSION FINDINGS

Table 1 Logistic regression model output for cancelled/postponed routine appointments

Routine Appointments	Odds ratio	Standard error	z	P>|z|	[95% conf. interval]	
**Country**						
UK	1.70	0.52	1.73	0.084	0.93	3.11
Germany	0.11	0.03	-7.8	0.000	0.06	0.19
Croatia	0.20	0.06	-5.62	0.000	0.11	0.35
Italy	0.62	0.20	-1.46	0.144	0.32	1.18
Belgium/Nether	0.16	0.05	-6.37	0.000	0.09	0.28
Other	0.34	0.09	-4.21	0.000	0.20	0.56
**Age**	1.32	0.17	2.19	0.029	1.03	1.69
**Education**	1.14	0.15	0.99	0.325	0.88	1.46
**CA type**						
Cleft Lip	1.03	0.22	0.15	0.883	0.68	1.55
Spina bifida	1.47	0.39	1.46	0.143	0.88	2.48
Down syndrome	1.85	0.41	2.78	0.005	1.20	2.86
DS + CHD	2.09	0.82	1.88	0.061	0.97	4.53
_cons	1.36	0.47	0.89	0.373	0.69	2.67

CHD – congenital heart defect; CA – congenital anomaly.
Comparator groups were Poland for country, and CHD for CA type

Table 2 Logistic regression model output for cancelled/postponed surgeries

**Surgeries**	Odds ratio	Standard error	z	P>|z|	[95% conf. interval]	
**Country**						
UK	0.91	0.27	-0.3	0.761	0.51	1.65
Germany	0.16	0.08	-3.68	0.000	0.06	0.43
Croatia	0.27	0.14	-2.6	0.009	0.10	0.73
Italy	0.21	0.12	-2.83	0.005	0.07	0.62
Belgium/Nether	0.34	0.14	-2.71	0.007	0.16	0.74
Other	0.25	0.10	-3.41	0.001	0.11	0.56
**Age**	0.83	0.14	-1.12	0.263	0.61	1.15
**Education**	1.33	0.20	1.83	0.068	0.98	1.79
**CA type**						
Cleft Lip	1.79	0.46	2.29	0.022	1.09	2.96
Spina bifida	1.19	0.42	0.49	0.622	0.60	2.38
Down syndrome	0.97	0.29	-0.09	0.927	0.54	1.75
DS + CHD	1.05	0.49	0.1	0.918	0.42	2.63
_cons	0.40	0.18	-2.06	0.04	0.17	0.96

CHD – congenital heart defect; CA – congenital anomaly.
Comparator groups were Poland for country, and CHD for CA type

Table 3 Logistic regression model output for cancelled/postponed test and procedures

**Tests/procedures**	Odds ratio	Standard error	z	P>|z|	[95% conf. interval]	
**Country**						
UK	1.08	0.27	0.3	0.767	0.66	1.74
Germany	0.11	0.04	-6.55	0.000	0.06	0.21
Croatia	0.29	0.09	-3.9	0.000	0.15	0.54
Italy	0.61	0.20	-1.51	0.132	0.33	1.16
Belgium/Nether	0.15	0.05	-5.31	0.000	0.07	0.30
Other	0.39	0.10	-3.69	0.000	0.23	0.64
**Age**	1.18	0.15	1.32	0.185	0.92	1.52
**Education**	1.15	0.14	1.16	0.246	0.91	1.47
**CA type**						
Cleft Lip	1.45	0.31	1.75	0.08	0.96	2.21
Spina bifida	2.78	0.74	3.86	0.000	1.65	4.67
Down syndrome	2.40	0.52	4.05	0.000	1.57	3.66
DS + CHD	1.69	0.60	1.47	0.142	0.84	3.41
_cons	0.65	0.23	-1.23	0.218	0.33	1.29

CHD – congenital heart defect; CA – congenital anomaly.
Comparator groups were Poland for country, and CHD for CA type

Table 4 Logistic regression model output for problems accessing medication (a little-very)

**Accessing medication**	Odds ratio	Standard error	z	P>|z|	[95% conf. interval]	
**Country**						
UK	1.42	0.36	1.39	0.165	0.87	2.32
Germany	0.14	0.07	-3.97	0.000	0.06	0.38
Croatia	0.08	0.06	-3.37	0.001	0.02	0.35
Italy	0.30	0.15	-2.38	0.017	0.11	0.81
Belgium/Nether	0.43	0.17	-2.17	0.030	0.20	0.92
Other	0.16	0.07	-4.07	0.000	0.07	0.39
**Age**	0.91	0.13	-0.65	0.518	0.69	1.21
**Education**	0.87	0.13	-0.95	0.340	0.65	1.16
**CA type**						
Cleft Lip	0.35	0.10	-3.66	0.000	0.20	0.61
Spina bifida	0.71	0.20	-1.23	0.218	0.41	1.23
Down syndrome	0.58	0.14	-2.23	0.026	0.36	0.94
DS + CHD	0.36	0.16	-2.37	0.018	0.15	0.84
_cons	1.24	0.49	0.56	0.578	0.58	2.68

CHD – congenital heart defect; CA – congenital anomaly.
Comparator groups were Poland for country, and CHD for CA type

Table 5 Logistic regression model output for face-to-face appointments re-scheduled as virtual

**Face-to-face rescheduled**	Odds ratio	Standard error	z	P>|z|	[95% conf. interval]	
**Country**						
UK	2.84	0.88	3.38	0.001	1.55	5.20
Germany	0.12	0.04	-7.17	0.000	0.07	0.21
Croatia	0.31	0.10	-3.76	0.000	0.17	0.57
Italy	0.21	0.07	-4.64	0.000	0.11	0.40
Belgium/Nether	0.18	0.06	-5.34	0.000	0.10	0.34
Other	0.41	0.10	-3.53	0.000	0.25	0.67
**Age**	1.15	0.15	1.07	0.284	0.89	1.48
**Education**	1.06	0.14	0.49	0.626	0.83	1.36
**CA type**						
Cleft Lip	1.37	0.29	1.51	0.131	0.91	2.07
Spina bifida	2.82	0.75	3.93	0.000	1.68	4.74
Down syndrome	3.29	0.71	5.47	0.000	2.14	5.03
DS + CHD	5.39	2.31	3.94	0.000	2.33	12.46
_cons	0.95	0.33	-0.16	0.873	0.48	1.88

CHD – congenital heart defect; CA – congenital anomaly.
Comparator groups were Poland for country, and CHD for CA type

Table 6 Logistic regression model output for the quality of virtual appointments (poor)

Quality of virtual appointments (poor)	Odds ratio	Standard error	z	P>|z|	[95% conf. interval]	
**Country**						
UK	0.44	0.12	-2.89	0.004	0.26	0.77
Germany	1.00†	(empty)				
Croatia	0.32	0.18	-1.99	0.047	0.11	0.98
Italy	0.62	0.35	-0.84	0.403	0.21	1.89
Belgium/Nether	0.09	0.09	-2.35	0.019	0.01	0.67
Other	0.46	0.18	-1.98	0.048	0.22	0.99
**Age**	0.95	0.15	-0.35	0.724	0.70	1.28
**Education**	0.56	0.09	-3.59	0	0.41	0.77
**CA type**						
Cleft Lip	0.72	0.21	-1.11	0.267	0.40	1.28
Spina bifida	0.65	0.22	-1.29	0.196	0.33	1.25
Down syndrome	0.71	0.19	-1.3	0.192	0.42	1.19
DS + CHD	0.65	0.29	-0.97	0.333	0.28	1.54
_cons	2.44	1.08	2.01	0.044	1.02	5.82

†0 participants in Germany rated virtual appointments as poor
CHD – congenital heart defect; CA – congenital anomaly. Comparator groups were Poland for country, and CHD for CA type

Table 6 Logistic regression model output for satisfaction with support from general practitioner

Support from GP	Odds ratio	Standard error	z	P>|z|	[95% conf. interval]	
**Country**						
UK	0.97	0.26	-0.1	0.920	0.57	1.65
Germany	16.28	6.08	7.47	0.000	7.83	33.86
Croatia	1.99	0.59	2.32	0.020	1.11	3.56
Italy	2.29	0.76	2.5	0.013	1.19	4.38
Belgium/Nether	4.05	1.33	4.27	0.000	2.13	7.69
Other	2.38	0.67	3.09	0.002	1.37	4.13
**Age**	0.90	0.12	-0.85	0.395	0.69	1.16
**Education**	0.95	0.12	-0.43	0.669	0.73	1.22
**CA type**						
Cleft Lip	0.76	0.18	-1.15	0.251	0.48	1.21
Spina bifida	1.38	0.38	1.16	0.247	0.80	2.36
Down syndrome	1.14	0.25	0.61	0.540	0.74	1.76
DS + CHD	1.45	0.56	0.97	0.333	0.68	3.07
_cons	0.46	0.17	-2.16	0.031	0.23	0.93

CHD – congenital heart defect; CA – congenital anomaly.
Comparator groups were Poland for country, and CHD for CA type

Table 7 Logistic regression model output for satisfaction with support from specialist doctor/nurse

Support from Specialist	Odds ratio	Standard error	z	P>|z|	[95% conf. interval]	
**Country**						
UK	1.38	0.33	1.32	0.188	0.86	2.21
Germany	12.10	4.46	6.76	0.000	5.87	24.92
Croatia	1.86	0.53	2.14	0.032	1.05	3.26
Italy	0.94	0.34	-0.17	0.868	0.46	1.92
Belgium/Nether	3.09	0.90	3.87	0.000	1.75	5.48
Other	1.75	0.45	2.18	0.029	1.06	2.89
**Age**	1.00	0.13	0.03	0.972	0.79	1.28
**Education**	1.04	0.13	0.34	0.735	0.82	1.34
**CA type**						
Cleft Lip	1.10	0.24	0.43	0.664	0.72	1.67
Spina bifida	0.94	0.25	-0.22	0.825	0.57	1.57
Down syndrome	0.55	0.12	-2.8	0.005	0.36	0.83
DS + CHD	1.06	0.39	0.16	0.876	0.51	2.18
_cons	0.49	0.17	-2.08	0.038	0.25	0.96

CHD – congenital heart defect; CA – congenital anomaly.
Comparator groups were Poland for country, and CHD for CA type

Table 8 Logistic regression model output for satisfaction with support from schools

Support from schools	Odds ratio	Standard error	z	P>|z|	[95% conf. interval]	
**Country**						
UK	2.40	0.80	2.63	0.009	1.25	4.61
Germany	1.76	0.90	1.11	0.267	0.65	4.81
Croatia	0.63	0.37	-0.79	0.431	0.19	2.01
Italy	1.96	0.74	1.78	0.076	0.93	4.11
Belgium/Nether	1.54	0.62	1.07	0.287	0.70	3.41
Other	1.32	0.49	0.75	0.455	0.64	2.75
**Age**	0.96	0.19	-0.23	0.822	0.65	1.41
**Education**	0.92	0.16	-0.46	0.645	0.66	1.30
**CA type**						
Cleft Lip	1.02	0.35	0.05	0.959	0.52	1.99
Spina bifida	1.37	0.50	0.85	0.393	0.67	2.81
Down syndrome	1.64	0.51	1.62	0.106	0.90	3.00
DS + CHD	1.53	0.89	0.74	0.459	0.49	4.76
_cons	0.37	0.21	-1.76	0.078	0.12	1.12

CHD – congenital heart defect; CA – congenital anomaly.
Comparator groups were Poland for country, and CHD for CA type

Table 9 Logistic regression model output for satisfaction with support from partner

**Support from partner**	Odds ratio	Standard error	z	P>|z|	[95% conf. interval]	
**Country**						
UK	1.62	0.42	1.87	0.062	0.98	2.70
Germany	3.61	1.62	2.86	0.004	1.50	8.70
Croatia	1.79	0.58	1.79	0.073	0.95	3.38
Italy	1.09	0.37	0.25	0.804	0.56	2.13
Belgium/Nether	0.84	0.24	-0.64	0.523	0.48	1.45
Other	1.01	0.27	0.02	0.981	0.60	1.69
**Age**	1.04	0.13	0.29	0.770	0.81	1.32
**Education**	1.01	0.13	0.06	0.954	0.79	1.28
**CA type**						
Cleft Lip	1.63	0.36	2.22	0.026	1.06	2.50
Spina bifida	1.70	0.48	1.88	0.061	0.98	2.97
Down syndrome	1.11	0.23	0.51	0.607	0.74	1.66
DS + CHD	1.15	0.41	0.38	0.702	0.57	2.32
_cons	1.71	0.59	1.53	0.125	0.86	3.37

CHD – congenital heart defect; CA – congenital anomaly.
Comparator groups were Poland for country, and CHD for CA type

Table 10 Logistic regression model output for satisfaction with support from friends and family

**Support from friends/family**	Odds ratio	Standard error	z	P>|z|	[95% conf. interval]	
**Country**						
UK	0.62	0.13	-2.22	0.027	0.40	0.95
Germany	1.77	0.55	1.81	0.070	0.96	3.27
Croatia	1.13	0.32	0.44	0.659	0.65	1.95
Italy	0.56	0.17	-1.89	0.059	0.30	1.02
Belgium/Nether	0.61	0.16	-1.86	0.062	0.36	1.03
Other	0.71	0.17	-1.44	0.151	0.44	1.14
**Age**	0.89	0.10	-1.08	0.280	0.71	1.10
**Education**	1.12	0.13	1.03	0.303	0.90	1.40
**CA type**						
Cleft Lip	1.15	0.23	0.71	0.478	0.78	1.69
Spina bifida	0.92	0.22	-0.34	0.731	0.57	1.48
Down syndrome	1.11	0.21	0.54	0.586	0.77	1.60
DS + CHD	1.17	0.38	0.47	0.636	0.62	2.21
_cons	1.56	0.48	1.43	0.151	0.85	2.86

CHD – congenital heart defect; CA – congenital anomaly.
Comparator groups were Poland for country, and CHD for CA type

Table 11 Logistic regression model output for satisfaction with support from other parents

Support from other parents	Odds ratio	Standard error	z	P>|z|	[95% conf. interval]	
**Country**						
UK	0.52	0.13	-2.6	0.009	0.32	0.85
Germany	0.77	0.32	-0.64	0.520	0.34	1.73
Croatia	0.89	0.27	-0.4	0.693	0.49	1.60
Italy	0.39	0.15	-2.47	0.013	0.18	0.82
Belgium/Nether	0.25	0.10	-3.45	0.001	0.11	0.55
Other	0.64	0.19	-1.54	0.122	0.36	1.13
**Age**	0.94	0.13	-0.47	0.636	0.71	1.23
**Education**	1.06	0.14	0.46	0.647	0.82	1.39
**CA type**						
Cleft Lip	1.60	0.39	1.93	0.054	0.99	2.57
Spina bifida	0.73	0.22	-1.03	0.303	0.41	1.32
Down syndrome	1.07	0.24	0.32	0.749	0.70	1.65
DS + CHD	1.25	0.49	0.57	0.572	0.58	2.68
_cons	1.84	0.69	1.64	0.101	0.89	3.82

CHD – congenital heart defect; CA – congenital anomaly.
Comparator groups were Poland for country, and CHD for CA type

Table 12 Logistic regression model output for satisfaction with support from patient organisations

Support from patient organisations	Odds ratio	Standard error	z	P>|z|	[95% conf. interval]	
**Country**						
UK	0.48	0.13	-2.76	0.006	0.28	0.81
Germany	1.14	0.52	0.28	0.780	0.46	2.81
Croatia	0.45	0.16	-2.21	0.027	0.22	0.91
Italy	0.39	0.15	-2.4	0.016	0.18	0.84
Belgium/Nether	0.12	0.08	-3.34	0.001	0.03	0.41
Other	0.49	0.15	-2.34	0.019	0.27	0.89
**Age**	0.96	0.15	-0.24	0.808	0.71	1.31
**Education**	0.99	0.15	-0.09	0.931	0.74	1.32
**CA type**						
Cleft Lip	1.46	0.40	1.4	0.162	0.86	2.50
Spina bifida	0.70	0.23	-1.07	0.284	0.36	1.34
Down syndrome	1.03	0.25	0.13	0.899	0.64	1.66
DS + CHD	1.26	0.51	0.57	0.572	0.57	2.81
_cons	1.33	0.55	0.69	0.488	0.59	2.99

CHD – congenital heart defect; CA – congenital anomaly.
Comparator groups were Poland for country, and CHD for CA type

Latos-Biele G6¶ ,Aet al. BMJ Open 2022; 12:e061428. doi: 10.1136/bmjopen-2022-061428

Table 13 Logistic regression model output for 'overall need for more support'

**Overall more support needed**	Odds ratio	Standard error	z	P>|z|	[95% conf. interval]	
**Country**						
UK	0.75	0.18	-1.18	0.236	0.47	1.21
Germany	0.55	0.17	-1.98	0.048	0.30	0.99
Croatia	0.38	0.14	-2.69	0.007	0.18	0.77
Italy	0.54	0.19	-1.72	0.086	0.27	1.09
Belgium/Nether	0.22	0.10	-3.38	0.001	0.09	0.53
Other	0.28	0.10	-3.59	0.000	0.14	0.56
**Age**	1.02	0.13	0.13	0.898	0.79	1.30
**Education**	0.92	0.11	-0.64	0.520	0.72	1.18
**CA type**						
Cleft Lip	0.53	0.13	-2.68	0.007	0.34	0.85
Spina bifida	0.86	0.23	-0.57	0.569	0.50	1.46
Down syndrome	1.03	0.21	0.14	0.889	0.69	1.54
DS + CHD	1.04	0.36	0.11	0.914	0.53	2.04
_cons	0.54	0.19	-1.76	0.078	0.28	1.07

CHD – congenital heart defect; CA – congenital anomaly.
Comparator groups were Poland for country, and CHD for CA type

Latos-Biele G6¶ ,A*et al. BMJ Open* 2022; 12:e061428. doi: 10.1136/bmjopen-2022-061428
