# Supplementary material for: COVID-19 and children with congenital anomalies: a European survey of parents’ experiences of healthcare services
Source: BMJ Open. 2022 Jul 19;12(7):e061428. doi: 10.1136/bmjopen-2022-061428 (PMC9301497; doi:10.1136/bmjopen-2022-061428)
Supplement: Author's manuscript [file bmjopen-2022-061428.draft_revisions.pdf]

BMJ Open

BMJ Open is committed to open peer review. As part of this commitment we make the peer review history of every article we publish publicly available.

When an article is published we post the peer reviewers' comments and the authors' responses online. We also post the versions of the paper that were used during peer review. These are the versions that the peer review comments apply to.

The versions of the paper that follow are the versions that were submitted during the peer review process. They are not the versions of record or the final published versions. They should not be cited or distributed as the published version of this manuscript.

BMJ Open is an open access journal and the full, final, typeset and author-corrected version of record of the manuscript is available on our site with no access controls, subscription charges or pay-per-view fees (http://bmjopen.bmj.com).

If you have any questions on BMJ Open's open peer review process please email info.bmjopen@bmj.com

BMJ Open

COVID-19 and children with congenital anomalies: a European survey of parents' experiences of healthcare services.

Journal:	*BMJ Open*
Manuscript ID	bmjopen-2022-061428
Article Type:	Original research
Date Submitted by the Author:	25-Jan-2022
Complete List of Authors:	Latos-Bieleńska, Anna; University of Medical Sciences, Department of Medical Genetics<br>Marcus, Elena; St George's University of London, Population Population Health Research Institute<br>Jamry-Dziurla, Anna; University of Medical Sciences, Department of Medical Genetics<br>Rankin, Judith; Newcastle University, Population Health Sciences Institute<br>Barisic, Ingeborg; Children's University Hospital of Zagreb, Centre of Excellence for Reproductive and Regenerative Medicine<br>Cavero- Carbonell, Clara; Fundacio per al Foment de la Investigacio Sanitaria i Biomedica, Rare diseases research unit<br>Den Hond, Elly; Provincial Institute for Hygiene<br>Garne, Ester; Hospital Lillebaelt, Kolding, Paediatric Department<br>Genard, Lucas; Provincial Institute for Hygiene<br>Santos, Ana ; National Health Institute Doutor Ricardo Jorge Department of Epidemiology<br>Lutke, Renee; University Medical Center Groningen, Department of Genetics<br>Dias, Carlos; National Institute of Health, Department of Epidemiology<br>Neergaard Pedersen, Christina; Hospital Lillebaelt, Kolding, Paediatric Department<br>Neville, Amanda; University de Ferrara, IMER Registry (Emilia Romagna Registry of Birth Defects)<br>Niemann, Annika; Medical Faculty Otto-von-Guericke University, Malformation Monitoring Centre Saxony-Anhalt<br>Odak, Ljubica; Children's Hospital Zagreb, Centre of Excellence for Reproductive and Regenerative Medicine, Medical School University of Zagreb<br>Páramo-Rodríguez, Lucía; Fundacio per al Foment de la Investigacio Sanitaria i Biomedica, Rare diseases research unit<br>Pierini, Anna; Institute of Clinical Physiology, Unit of Epidemiology of Rare Diseases and Congenital Anomalies<br>Rissmann, Anke; Medical Faculty Otto-von-Guericke University, Malformation Monitoring Centre Saxony-Anhalt<br>Morris, Joan; St George's, University of London, Population Health Research Institute
Keywords:	COVID-19, PAEDIATRICS, International health services < HEALTH

	SERVICES ADMINISTRATION & MANAGEMENT

SCHOLARONE™
Manuscripts

I, the Submitting Author has the right to grant and does grant on behalf of all authors of the Work (as defined in the below author licence), an exclusive licence and/or a non-exclusive licence for contributions from authors who are: i) UK Crown employees; ii) where BMJ has agreed a CC-BY licence shall apply, and/or iii) in accordance with the terms applicable for US Federal Government officers or employees acting as part of their official duties; on a worldwide, perpetual, irrevocable, royalty-free basis to BMJ Publishing Group Ltd ("BMJ") its licensees and where the relevant Journal is co-owned by BMJ to the co-owners of the Journal, to publish the Work in this journal and any other BMJ products and to exploit all rights, as set out in our licence.

The Submitting Author accepts and understands that any supply made under these terms is made by BMJ to the Submitting Author unless you are acting as an employee on behalf of your employer or a postgraduate student of an affiliated institution which is paying any applicable article publishing charge ("APC") for Open Access articles. Where the Submitting Author wishes to make the Work available on an Open Access basis (and intends to pay the relevant APC), the terms of reuse of such Open Access shall be governed by a Creative Commons licence – details of these licences and which Creative Commons licence will apply to this Work are set out in our licence referred to above.

Other than as permitted in any relevant BMJ Author's Self Archiving Policies, I confirm this Work has not been accepted for publication elsewhere, is not being considered for publication elsewhere and does not duplicate material already published. I confirm all authors consent to publication of this Work and authorise the granting of this licence.

For peer review only

COVID-19 and children with congenital anomalies: a European survey of parents' experiences of healthcare services.

Anna Latos-Bielenska[1]*, Elena Marcus[2]*, Anna Jamry-Dziurla[1], Judith Rankin[3], Ingeborg Barišić[4], Clara Cavero-Carbonell[5], Elly Den Hond[6], Ester Garne[7], Lucas Genard[6], Ana João Santos[8], L Renée Lutke[9], Carlos Matias Dias[8], Christina Neergaard Pedersen[7], Amanda J Neville[10], Annika Niemann[11], Ljubica Odak[4], Lucía Páramo-Rodríguez[5], Anna Pierini[12], Anke Rissmann[11], Joan K Morris[2].

[1]Chair and Department of Medical Genetics, Poznan University of Medical Sciences, Collegium Maius, Fredry 10, 61-701, Poznań, Poland.

Anna Latos-Bielenska, professor.

Anna Jamry-Dziurla, deputy registry co-ordinator.

[2]Population Health Research Institute, St George's, University of London, Cranmer Terrace, London SW17 0RE, United Kingdom.

Elena Marcus, postdoctoral researcher.

Joan K Morris, professor of statistics.

[3]Population Health Sciences Institute, Newcastle University, Newcastle upon Tyne, NE1 7RU, United Kingdom.

Judith Rankin, professor of maternal and child health.

[4]Children's Hospital Zagreb, Centre of Excellence for Reproductive and Regenerative Medicine, Medical School University of Zagreb, Ul. Vjekoslava Klaića 16, 10000, Zagreb, Croatia.

Ingeborg Barišić, professor.

Ljubica Odak, consultant paediatrician.

[5]Rare Diseases Research Unit, Foundation for the Promotion of Health and Biomedical Research in the Valencian Region, Av. de Catalunya, 21, 46020 València, Spain.

Clara Cavero-Carbonell, researcher and head of the Rare Diseases Research Unit.

Lucía Páramo-Rodríguez, research assistant.

[6]Provincial Institute for Hygiene (PIH), Kronenburgstraat 45, 2000 Antwerpen, Belgium.

Elly Den Hond, senior research associate.

For peer review only - http://bmjopen.bmj.com/site/about/guidelines.xhtml

For peer review only

Lucas Genard, researcher.

[7]University Hospital Lillebaelt, Sygehusvej 24, 6000 Kolding, Denmark.

Ester Garne, consultant pediatrician and associate professor.

Christina Neergaard Pedersen, specialty registrar.

[8]Department of Epidemiology, National Institute of Health Doctor Ricardo Jorge, Av. Padre Cruz, 1600-609 Lisboa, Portugal.

Ana João Santos, senior technician.

Carlos Matias Dias, department co-ordinator.

[9]Department of Genetics, University Medical Center, University of Groningen, 9712 CP Groningen, Netherlands.

L Renée Lutke, pharmacist/researcher

[10] IMER Registry (Emilia Romagna Registry of Birth Defects), Center for Clinical and Epidemiological Research, University of Ferrara, Azienda Ospedaliero- Universitaria di Ferrara, Corso Giovecca, 203, 44121 Ferrara (Italy).

Amanda J Neville, EUROCAT registry lead.

[11]Malformation Monitoring Centre Saxony-Anhalt, Medical Faculty, Otto-von-Guericke-University Magdeburg, Leipziger Str. 44, 39120 Magdeburg, Germany.

Anke Rissmann, consultant paediatrician and registry leader.

Annika Niemann, research associate.

[12]Unit of Epidemiology of Rare Diseases and Congenital Anomalies, Institute of Clinical Physiology, National Research Council, Via Giuseppe Moruzzi, 1, 56124 Pisa, Italy.

Anna Pierini, senior researcher.

*****Joint first author: these authors contributed equally

Corresponding author: Dr Elena Marcus, Population Health Research Institute, St George's, University of London, Cranmer Terrace, London SW17 0RE, UK. Email: emarcus@sgul.ac.uk.

Word Count: 3,946

For peer review only - http://bmjopen.bmj.com/site/about/guidelines.xhtml

ABSTRACT

Objective

To survey parents and carers of children with a congenital anomaly (CA) across Europe about their child's experiences of healthcare services, and their experiences of support during the COVID-19 pandemic.

Design

Cross-sectional study.

Setting

Online survey in 10 European countries, open from 8th March 2021 to 14th July 2021.

Population

1,070 parents and carers of children aged 0-10 years with a cleft lip, spina bifida, congenital heart defect (CHD) requiring surgery, and/or Down syndrome.

Main outcome measures

Parental views about: the provision of care for their child (cancellation/postponement of appointments, virtual appointments, access to medication), the impact of disruptions to healthcare on their child's health and well-being, and satisfaction with support from medical sources, organisations and close relationships.

Results

Disruptions to healthcare appointments were significantly higher ($p<0.001$) in the UK and Poland, with approximately two-thirds of participants reporting 'cancelled or postponed' tests (67/101; 256/389) and procedures compared with approximately 20% in Germany (13/74) and Belgium/Netherlands (11/55). A third of participants in the UK and Poland reported 'cancelled or postponed' surgeries (22/72; 98/266) compared with only 8% in Germany (5/64). In Poland, 43% (136/314) of parents reported that changes to their child's ongoing treatment had moderately to severely affected their child's health, significantly higher than all other countries ($p<0.001$). Satisfaction ratings for support from general practitioners were lowest in the UK and Poland, and lowest in Poland and Italy for specialist doctors and nurses.

Conclusion

A large proportion of participants reported disruptions to healthcare during the pandemic, which for some had a significant impact on their child's health. Regional differences in disruptions raise questions about the competence of certain healthcare systems to meet the needs of this vulnerable group of patients and indicate improvements should be strived for in some regions.

Keywords

congenital anomaly, COVID-19, child, parental experience, provision of healthcare, support, survey

For peer review only - http://bmjopen.bmj.com/site/about/guidelines.xhtml

STRENGTHS AND LIMITATIONS

- Surveys the experiences of a large total number of parents and carers across multiple European countries and congenital anomaly types. The proportion of each CA type in the study sample reflects the relative number of live births with each CA in Europe.
- High completion rates and item-level response rates, suggests that survey items were relevant to participants and easy to complete.
- Potential bias in responses due to the use of social media for recruitment, for example, excluding people living with 'digital poverty' and those who do not engage with patient and parent organisations. The experiences of this sample may differ to the wider population of parents and carers.
- Inability to conduct a full pilot of the final survey to explore item acceptability, comprehension, and relevance. Possible that there may be some issues with the wording or content of items.

BACKGROUND

The coronavirus disease 2019 (COVID-19) pandemic put pressure on healthcare systems worldwide, causing severe disruptions to the delivery of non-essential services, as staff were re-deployed to acute care, and outpatient treatment and follow-up was reduced due to concerns about viral transmission in hospital.(1-3) Non-urgent elective care was the most heavily impacted, with a record backlog of 5.6 million cases reported in England in July 2021.(4, 5)

Congenital anomalies (CAs) are a range of conditions that are present from birth and remain a leading cause of childhood morbidity and long-term disability.(6, 7) Children with CAs require regular clinical follow-up,(8) including more frequent primary care appointments and hospital admissions than children without CAs.(9-11) Although children are less affected by SARS-CoV-2 infection than adults,(12) Down syndrome has been indicated as a risk factor of severe disease and mortality,(13, 14) and children with underlying conditions may be at increased risk of infection.(15) It is crucial to document the healthcare experiences of children with CAs during this period of increased pressure on healthcare systems, especially as they represent a vulnerable population. Yet, at present, there is limited published research investigating the impact of the COVID-19 pandemic on the provision of care for children with CAs. Existing research, conducted during the first wave of the pandemic in 2020, suggests a high proportion of cancellations to children's healthcare appointments and treatments.(16-18) Disruptions to paediatric services were found to cause anxiety for parents,(17) and fear that their child's health may be negatively affected.(18) Parents also reported a lack of

For peer review only - http://bmjopen.bmj.com/site/about/guidelines.xhtml

support from healthcare professionals, including the absence of specific COVID-19 related guidance for children.(18)

This paper describes a cross-sectional online survey which explored the views of parents and carers of children with CAs about: (a) their child's healthcare experiences, and (b) their experiences of support, one year into the pandemic. The survey was conducted as part of a collaborative European project, "Establishing a linked European Cohort of Children with CAs (EUROlinkCAT)", which aims to investigate health and educational outcomes in children born with CAs using population-based data. Due to differences in the level of restrictions, healthcare systems, and the availability of resources between countries, the survey was conducted in multiple countries across Europe, to explore any possible variations in the provision of care. The survey therefore aimed to compare and contrast the experiences of caregivers, and identify best practices that may help address current challenges and inform health care planning for future pandemics or crisis situations.

METHODS

This study is reported following the Strengthening the Reporting of Observational studies in Epidemiology (STROBE) guidelines.(19) The findings presented are a subsection of a cross-sectional online survey, conducted by the EUROlinkCAT team, which explored the wider information and support needs of parents and carers of children with CAs in 10 European countries. This paper focuses on the healthcare experiences and health status of children during the COVID-19 pandemic, and parent and carer experiences of support. The survey was launched in the United Kingdom (UK) and Poland on 8th March 2021 and kept open until 14th July 2021. Ethical approval for the study was granted by the St George's (University of London) Research Ethics Committee on 18th December 2020 (reference number: 2020.0311). Local ethical approvals (or evidence that no further approvals were required) were obtained from each participating country and the survey was launched in a staggered manner in each country, as and when translations were finalised and approvals granted (Table 1).

For peer review only - http://bmjopen.bmj.com/site/about/guidelines.xhtml

Table 1 Recruitment period and participant characteristics by country group.

Characteristic	All	UK	Poland	Germany	Croatia	Italy	Belgium/ Netherlands	Other EU†
**Recruitment period**								
Start date	-	8 Mar 2021	8 Mar 2021	11 May 2021	26 Apr 2021	16 Jun 2021	19 Apr 2021	6 Apr 2021
End date	-	14 Jul 2021	14 Jul 2021	14 Jul 2021	14 Jul 2021	31 Jul 2021	14 Jul 2021	14 Jul 2021
**N**	986	120	476	97	68	59	74	92
**Age**								
≤30	162 (17%)	18 (15%)	93 (20%)	13 (13%)	8 (12%)	4 (7%)	15 (20%)	11 (12%)
31-40	516 (53%)	53 (45%)	264 (56%)	51 (53%)	37 (55%)	27 (46%)	35 (47%)	49 (53%)
>40	301 (31%)	47 (40%)	115 (24%)	33 (34%)	22 (33%)	28 (47%)	24 (32%)	34 (35%)
**Relation to child**								
Mother	911 (92%)	116 (97%)	449 (94%)	81 (84%)	63 (93%)	52 (88%)	64 (86%)	86 (95%)
Father	65 (7%)	2 (2%)	24 (5%)	13 (13%)	5 (7%)	6 (10%)	10 (14%)	5 (5%)
Other‡	8 (1%)	1 (1%)	3 (1%)	3 (3%)	-	1 (2%)	-	-
**Employment**								
Employed	586 (60%)	81 (68%)	223 (47%)	61 (62%)	54 (79%)	44 (75%)	61 (82%)	62 (69%)
Homemaker/carer	301 (31%)	36 (30%)	198 (42%)	27 (29%)	7 (10%)	11 (19%)	8 (11%)	14 (16%)
Other*	94 (9%)	3 (3%)	52 (11%)	9 (9%)	7 (10%)	4 (7%)	5 (7%)	14 (16%)
**Education**								
School ≤18 years	390 (40%)	44 (37%)	163 (35%)	61 (67%)	19 (28%)	30 (52%)	44 (60%)	29 (32%)
University	482 (49%)	50 (42%)	257 (53%)	27 (29%)	45 (66%)	19 (33%)	29 (39%)	55 (60%)
Post-graduate	106 (11%)	25 (21%)	56 (11%)	3 (3%)	4 (6%)	9 (16%)	1 (1%)	8 (9%)
**Migrant status**								
>10 years/from birth	924 (94%)	111 (93%)	467 (98%)	86 (88%)	64 (94%)	50 (86%)	71 (96%)	75 (81%)
6-10 years	30 (3%)	5 (4%)	5 (1%)	6 (7%)	2 (3%)	4 (7%)	1 (1%)	7 (8%)
1-5 years	28 (3%)	4 (3%)	2 (0.4%)	5 (5%	2 (3%)	4 (7%)	2 (3%)	9 (10%)
<1 year	2 (0.2%)	-	1 (0.2%)	-	-	-	-	1 (1%)

†Other European countries: Denmark (n=39), Portugal (n=23), Spain (n=16), Ireland (n=5), Bulgaria (n=2), Albania (n=1), Cyprus (n=1), Lithuania (n=1), Norway (n=1), Romania (n=1), Sweden (n=1), Ukraine (n=1).
‡Other family member (n=3), legal guardian related to the child (n=2), legal guardian unrelated to the child (n=3).
*Unemployed (n=56), long-term sick/disabled (n=17), on furlough (n=12), student (n=8), retired (n=1)

For peer review only

For peer review only - http://bmjopen.bmj.com/site/about/guidelines.xhtml

Participants

The survey was open to parents, carers, and guardians (termed henceforth as *parents*) of children up to 10 years of age who have one or more of the following CAs: cleft lip (with or without a cleft palate), spina bifida, congenital heart defect (CHD) which required surgery, and Down syndrome. Due to the high level of heterogeneity across all CAs, these groups were pre-defined and selected to cover different types of impairments, with likely differing impacts on the experiences of the child and parent: (a) physical disability (spina bifida), (b) learning disability (Down syndrome), (c) visible defects (cleft lip), and (d) non-visible defects (CHD). Participants were actively recruited in 10 European countries: Belgium, Croatia, Denmark, Germany, Italy, Netherlands, Poland, Portugal, Spain, and the UK.

Recruitment

Participants were recruited with convenience sampling which was conducted online via social media (Twitter and Facebook), charities and patient organisations within each participating country (e.g. the Down Syndrome Association in the UK), and closed support groups on Facebook. Potential participants were provided with a link to the survey website which included all language versions of the survey. Participants were provided with the participant information sheet at the start of the survey, and depending on local ethical requirements, participants were either required to complete an online consent form or consent was implied by completion of the survey. As the survey was shared across online platforms and by a number of international organisations, responses were also received from parents living in other European countries (e.g. Ireland), and these were retained in the analysis.

Survey

The content of the survey was developed following a literature review, and input from expert clinicians, parents and educators, academics with expertise in CA research and questionnaire development, and a Public Involvement and Community Engagement (PICE) lead. The survey included the following sections: (1) Parent Demographics (9 items), (2) Child Demographics and Medical Information (7 items), (3) Provision of Healthcare (7 items), (4) Impact on the Child (4 items), and (5) Support for Parents (2 items). Response options varied and comprised: Yes-No (Provision of Healthcare), Not at all-A little-Quite a bit-Very much (Provision of Healthcare), Not at all satisfied-A little satisfied-Quite satisfied-Very satisfied (Support for Parents), and Much worse-Somewhat worse-About the same-Somewhat better-Much better (Impact on Child). All items were close-ended, therefore quantitative data were collected only. In relation to the timeframe,

For peer review only - http://bmjopen.bmj.com/site/about/guidelines.xhtml

participants were asked to reflect on their experiences from the start of the pandemic in January 2020 to the time at which they completed the survey.

Translation

The survey was developed in English and translated into eight European languages following existing guidance.(20) The Dutch version was used in Belgium and the Netherlands. The survey was initially translated into Polish and Italian to check for any translatability issues, and relevant amendments were subsequently made to the English version accordingly. These languages were selected because they have different origins (Slavic and Romance) with differing translation issues, and the research team included native Polish (ALB, AJ) and native English-Italian bilingual (EM) speakers. Translations were carried out in four steps for each language version: (1) a native speaker of the target language with good command of English conducted the initial translation, (2) the translation was checked by at least one other native speaker of the target language and any problems discussed and reconciled, (3) the survey was back-translated by a native English speaker (or person with a good command of the English language), and who was naïve to the original version, (4) the back-translated survey was reviewed by EM against the original English language version and any semantic or conceptual discrepancies in the back-translation were flagged and discussed with the translators until they were resolved. Due to differences in education systems across Europe, equivalent terminology for participants' education level was not available, and categories were selected to reflect local education systems within each country.

Data collection

Study data were collected and managed using Research Electronic Data Capture(21) (REDCap) tools hosted at St George's, University of London. All data collected were anonymous and it was therefore not possible to verify CA diagnoses. To keep the survey fully anonymous no internet protocol (IP) addresses were collected, so it was not possible to prevent multiple participation. Participants were initially allowed to skip any item, however, following an interim analysis on 27th April, a high proportion of missing data for country and CA type was noted. As these data were crucial to the research question, these two items were subsequently made mandatory.

Patient and Public Involvement

People with experience in caring for or teaching children with CAs contributed to the development of the survey. These were (a) three parents of children with CAs who also run patient organisations/charities relevant to their child's condition (Down syndrome, spina bifida, metachromatic leukodystrophy), (b) a clinical geneticist who works closely with a number of parent organisations, and (c) a teacher of children with special educational needs and disabilities. Each

For peer review only - http://bmjopen.bmj.com/site/about/guidelines.xhtml

individual commented on an early draft of the survey, including the overall content of the survey, and the wording of questions and response options. This feedback was reviewed by the research team and relevant modifications were made to the draft survey to address it.

Findings from the study will be shared with members of the public, parents and carers, healthcare professionals and relevant stakeholders via scientific publications, lay reports, social media and conferences.

Data analysis

Descriptive statistics were conducted using Stata 17.0 software.(22) Data were checked to ensure that answers were consistent (e.g. identifying if a mother replying that she is 20-25 years old is retired). Outcomes scored on 4-point Likert scales (very much/satisfied vs. other responses) were dichotomised and outcomes scored on a 5-point Likert scale were collapsed into three categories by merging the two lowest response options (much worse, somewhat worse) and the two highest response options (much better, somewhat better). Data were modelled using multivariate logistic regressions and ordinal logistic regressions which included the child's anomaly type, and parent's country of residence, age and education level. The impact of country and anomaly type on outcomes was explored, choosing the largest categories as the comparator groups (Poland and CHD). For age and education, categorical data were collected. For the analysis each variable was re-coded into three groups: age (<30 years; 31-40 years, >40 years), education (formal education/technical training until 16 or 18 years; university degree; post-graduate degree). Age and education were included in our regression models as ordinal variables. To control for multiple comparisons, the alpha level was adjusted to $p < 0.01$ for all analyses. It was unlikely that data were missing at random so more sophisticated multiple imputation techniques were not adopted.

We aimed to recruit 80 participants per country which would have resulted in a power of 80% to determine that a country with 20% of participants replying category 4 (very much/satisfied) was statistically significantly different at the 95% level of significance from a country with 40% of participants replying category 4. Owing to delays in obtaining ethical approvals, this target was not met within the timescales for some countries. Data were presented by country if these were available for at least 50 participants. Where there were <50 participants, data were combined into an 'other European country' group (termed henceforth as *Other EU*), which included participants from a heterogenous group of countries. Due to similarities in survey responses, geographical location, and language, data for Belgium (n=46) and the Netherlands (n=28) were combined into a single group. For CAs, data were categorised according to the four anomalies, and a separate category created for children with Down syndrome and a CHD, a common co-morbidity.(23) There

For peer review only - http://bmjopen.bmj.com/site/about/guidelines.xhtml

were too few participants to create meaningful categories for children who had other combinations of the four anomalies (n=15), and these were excluded from the analysis.

Given that a multi-modal online recruitment strategy was used, it was not possible to estimate how many potential participants the survey reached in order to calculate response rates.(24) Completion rates (number of participants who started the survey/number who completed the survey)(25) and item-level response rates (proportion of participants completing each item)(26) are reported.

RESULTS

Participant characteristics

1,109 parents across Europe completed the survey, of which 986 (89%) were included in the analysis. Participants were excluded from the analysis if: country data were missing (n=80), CA data were missing (n=24), or if participants were from non-European countries (n=4). Fifteen participants with mixed combinations of the four anomaly types were also excluded. The overall completion rate was 84%, which ranged from 78% in Italy to 92% in Germany and Belgium. Item-level response rates ranged from 96% to 99% across outcome variables.

Participants lived in Poland (n=476), the UK (n=120), Germany (n=97), Belgium/Netherlands (n=74), Croatia (n=68), Italy (n=59). The Other EU group (n=92) comprised participants from: Denmark (n=39), Portugal (n=23), Spain (n=16), Ireland (n=5), Bulgaria (n=2), Albania (n=1), Cyprus (n=1), Lithuania (n=1), Norway (n=1), Romania (n=1), Sweden (n=1), Ukraine (n=1). Most respondents were mothers (92%), aged 31-40 years (71%), and in full- or part-time employment (59%) (Table 1). In terms of education, 40% of participants had received formal education up to 16 or 18 years or technical training, 49% had a university degree, and 11% a post-graduate degree. Few participants had lived in their country of residence for <10 years (6%).

Child characteristics

The largest CA group was CHD (n=327; 33%). Other children were diagnosed with Down syndrome (n=262; 26%), a cleft lip (n=230; 23%), spina bifida (n=112; 11%) and Down syndrome with a CHD (n=55; 6%). In terms of co-morbidities, 25% of children had another CA, and 43% had another health condition. The most common age category was 1-3 years (35%) and there was a slightly higher proportion of male children (56%). Just over a third of children attended school (36%), whereas 62% were not yet of school age, and 2% were either home-schooled or unable to be schooled due to their health status.

For peer review only

(a) Provision of healthcare across countries

Cancelled or postponed appointments

Cancellations or postponements of routine appointments were reported by 68% (623/920) of the whole sample, by 53% (427/803) for planned tests or procedures, and by 26% (121/609) for planned surgeries. The UK and Poland had the largest proportions of parents reporting cancelled or postponed appointments for each category (Figure 1). For routine appointments and planned tests/procedures, proportions were significantly lower in Germany, Croatia, Belgium/Netherlands, and the Other EU group compared with Poland (Table 2). For planned surgeries, all countries except the UK had a significantly lower proportion of cancelled or postponed appointments than Poland.

Table 2 Proportion of participants reporting 'cancelled or postponed' routine appointments, planned tests or procedures, and planned surgeries, by country.

Country	Routine appointments (N†=920)		Planned tests or procedures (N†=803)		Planned surgeries (N†=609)	
	Unadjusted % [95% CI]	*Adjusted** % [95% CI]	*Unadjusted* % [95% CI]	*Adjusted** % [95% CI]	*Adjusted** % [95% CI]	*Unadjusted* % [95% CI]
UK	88 [82, 94]	86 [80, 93]	67 [58, 76]	67 [57, 76]	33 [22, 44]	31 [20, 41]
Poland	79 [75, 83]	79 [75, 83]	66 [61, 70]	65 [60, 70]	35 [29, 41]	37 [31, 43]
Germany	29 [19, 38]	31 [21, 42]	16 [8, 24]	18 [9, 27]	8 [1, 15]	7 [1, 14]
Croatia	46 [34, 58]	44 [32, 56]	36 [23, 49]	36 [23, 49]	13 [3, 24]	14 [3, 24]
Italy	69 [57, 81]	70 [58, 82]	52 [38, 66]	54 [40, 68]	11 [0, 20]	9 [1, 18]
Belgium/Netherlands	34 [23, 46]	39 [27, 51]	20 [9, 31]	23 [11, 34]	16 [6, 25]	17 [7, 27]
Other EU	56 [46, 67]	57 [47, 67]	43 [32, 53]	43 [32, 53]	12 [4, 20]	12 [4, 19]

†Total number of participants excluding 'not applicable' responses. Missing data: routine appointments (9 participants), planned tests or procedures (8 participants), planned surgeries (5 participants).
*Adjusted by congenital anomaly type, parental age, and education level.
CI = confidence intervals

Virtual appointments (by telephone or online)

Overall, 61% (544/891) of participants reported that their child's face-to-face appointments had been rescheduled as virtual appointments. This proportion was highest in the UK (87%), significantly higher than in Poland (71%). In all other countries this proportion was significantly lower than Poland (Table 3).

For peer review only - http://bmjopen.bmj.com/site/about/guidelines.xhtml

Table 3 Proportion* of participants reporting appointments rescheduled as virtual, virtual appointments rated as 'poor', and problems accessing medication, by country.

Country	Appointments rescheduled as virtual (N†=891)		Virtual appointments rated as 'poor' (N†=552)		Problems accessing medication (N†=713)	
	Unadjusted % [95% CI]	*Adjusted** % [95% CI]	*Unadjusted* % [95% CI]	*Adjusted** % [95% CI]	*Unadjusted* % [95% CI]	*Adjusted** % [95% CI]
UK	87 [81, 93]	87 [81, 93]	21 [13, 29]	21 [13, 29]	43 [33, 54]	42 [32, 52]
Poland	72 [68, 76]	71 [67, 75]	37 [32, 42]	37 [32, 43]	34 [29, 39]	34 [29, 39]
Germany	24 [15, 33]	25 [16, 35]	0 [0, 17]	0 [0, 17]	6 [1, 12]	7 [1, 14]
Croatia	46 [33, 59]	46 [33, 58]	15 [2, 29]	17 [2, 31]	5 [0, 11]	4 [0, 10]
Italy	34 [21, 47]	37 [23, 50]	29 [8, 51]	27 [7, 48]	14 [3, 25]	14 [3, 25]
Belgium/Netherlands	28 [17, 40]	34 [22, 46]	6 [0, 18]	5 [0, 15]	19 [8, 29]	19 [8, 29]
Other EU	50 [40, 60]	52 [42, 62]	23 [10, 35]	22 [10, 34]	9 [2, 16]	8 [2, 14]

†Total number of participants excluding 'not applicable' responses. Missing data: appointments rescheduled as virtual (8 participants), virtual appointments rated as 'poor' (11 participants), problems accessing medication (8 participants).

*Adjusted by congenital anomaly type, parental age and education level.

CI = confidence intervals

Overall, 29% (159/541) of participants reported their child's virtual appointments as being of 'poor' quality overall. This proportion was highest in Poland (37%), and significantly lower in the UK (21%), Belgium/Netherlands (5%), and Germany (0%) (Table 3). There was a significant impact of education level on ratings, whereby more highly educated participants were less likely to rate the overall quality of their virtual appointments as 'poor' (Odds Ratio [OR] = 0.55, 95% CI: 0.41-0.77; p=0.000).

Access to medication

Overall, 26% (182/705) of participants reported some problems accessing medication for their child during the pandemic. This proportion was highest in the UK (42%) and Poland (34%) (Table 3). Italy (14%), the Other EU group (8%), Germany (7%), and Croatia (4%) all had significantly fewer participants reporting problems compared with Poland (Table 3).

(b) Impact on the child's health and wellbeing across countries

Overall, 30% (221/749) of participants reported that changes to their child's treatment during the pandemic had moderately to severely compromised their child's health. This figure was significantly higher in Poland (43%) compared with the UK (28%), the Other EU group (20%), Croatia (15%), Italy (12%), Germany (12%), and Belgium/Netherlands (7%) (Figure 2).

The majority of participants rated their child's physical health (68%; 634/927) and emotional wellbeing (56%; 515/927) as being 'about the same' as it was prior to the pandemic. Overall, there

For peer review only - http://bmjopen.bmj.com/site/about/guidelines.xhtml

was a greater proportion of participants who rated their child's emotional wellbeing as 'worse' (35%; 319/927) compared to 'worse' ratings for physical health (17%; 162/927).

There was a significant impact of country on ratings for physical health, with all countries less likely to rate their child's physical health as 'worse' than before the pandemic compared with Poland (Figure 3). Ratings for the impact of COVID-19 on emotional wellbeing were similar across countries.

(c) Support for parents across countries

Overall, 23% (220/957) of participants reported that they would have liked more support during the pandemic 'very much'. This proportion was highest in Poland (30%), and significantly lower in Croatia (14%), Belgium/Netherlands (9%), and the Other EU group (11%) (Figure 4). In terms of the source of support, satisfaction ratings were lowest for support from medical sources and organisations, and highest for people that participants had close relationships with, such as their partner (Table 4).

For peer review only

For peer review only - http://bmjopen.bmj.com/site/about/guidelines.xhtml

Table 4 Proportion* of participants reporting that they were 'very satisfied' with the support they received from each source, by country.

Country	GP (N†=775)	Specialist doctor/nurse (N†=792)	Partner (N†=868)	Friends/family (N†=883)	Parents of children with same condition (N†=637)	Patient organisations (N†=510)	Schools (N†=369)
	% [95% CI]	% [95% CI]	% [95% CI]	% [95% CI]	% [95% CI]	% [95% CI]	% [95% CI]
UK	25 [16, 34]	39 [29, 49]	79 [71, 86]	49 [40, 58]	51 [41, 61]	38 [27, 48]	47 [34, 60]
Poland	26 [21, 30]	34 [27, 37]	69 [65, 74]	61 [56, 66]	66 [61, 71]	56 [50, 62]	27 [20, 34]
Germany	85 [76, 94]	84 [76, 93]	89 [81, 97]	73 [62, 85]	60 [42, 79]	59 [38, 80]	39 [17, 61]
Croatia	41 [28, 53]	46 [34, 59]	80 [1, 90]	64 [52, 75]	64 [51, 76]	36 [21, 51]	19 [2, 36]
Italy	44 [29, 59]	31 [16, 45]	71 [58, 84]	47 [32, 61]	44 [26, 61]	33 [18, 49]	42 [27, 57]
Belgium/ Netherlands	57 [44, 72]	59 [46, 71]	66 [54, 77]	49 [37, 61]	33 [17, 50]	14 [0, 27]	36 [20, 52]
Other EU	45 [33, 57]	45 [34, 56]	70 [60, 79]	52 [42, 63]	56 [43, 68]	38 [26, 51]	33 [19, 46]
Total	37%	42%	72%	58%	60%	46%	34%

*Adjusted by congenital anomaly type, parental age, and education level. Unadjusted proportions are not included in this table.
†Total number of participants completing the item, excluding 'not applicable' responses. Missing data: GP (n=10), specialist doctor/nurse (n=16), partner (n=11), friends/family (n=14), parents of children with same condition (n=17), patient organisations (n=18), schools (n=35).
CI = confidence interval; GP = general practitioner

For peer review only

Medical sources

The UK and Poland had the lowest proportion of 'very satisfied' ratings for GPs, 25% and 26% respectively (Table 4). Compared to Poland, ratings were significantly higher in Germany (85%) and the Other EU group (45%). Italy and Poland had the lowest 'very satisfied' ratings for specialist doctors/nurses, 31% and 32%, respectively. Compared to Poland, ratings were significantly higher in Germany (84%) and the Other EU group (45%) (Table 4).

Organisations

The highest proportion of 'very satisfied' ratings for patient organisations were for parents in Germany (59%) and Poland (56%) (Table 4). Compared with Poland, these satisfaction ratings were significantly lower in the UK (38%) and Belgium/Netherlands (14%). The UK had the highest proportion of participants who were 'very satisfied' with support from their child's school (47%), however, there were no significant country related effects.

Close relationships

Poland had the highest proportion of 'very satisfied' ratings for support from parents of other children with the same health condition (66%), significantly higher than the UK (51%) and Belgium/Netherlands (33%) (Table 4). There were no significant differences in satisfaction ratings for support from 'partner' or 'friends/family' across countries.

(d) Outcomes across CA types

There were few differences across CA types, with significant differences only found for items relating to the provision of healthcare. In summary, parents of children with CHD (43%) reported a significantly lower proportion of 'cancelled or postponed' tests/procedures compared with parents of children with spina bifida (65%) and Down syndrome (alone) (62%) (Table 5). The CHD group also reported significantly fewer rescheduled appointments (49%) compared with the Down syndrome with CHD (80%), Down syndrome (72%), and spina bifida (70%) groups (Table 5). A lower proportion of parents of children with a cleft lip (17%) reported problems accessing medication compared with the CHD group (34%) (Table 5).

For peer review only - http://bmjopen.bmj.com/site/about/guidelines.xhtml

Table 5 Proportion of participants reporting 'cancelled or postponed' planned tests/procedures, appointments rescheduled as virtual, virtual appointments rated as 'poor', and problems accessing medication, by CA type.

**Congenital anomaly**	**Planned tests or procedures (N†=803)**		**Appointments rescheduled as virtual (N†=891)**		**Virtual appointments rated as 'poor' (N†=552)**		**Problems accessing medication (N†=713)**	
	Unadjusted % [95% CI]	*Adjusted** % [95% CI]	*Unadjusted* % [95% CI]	*Adjusted** % [95% CI	*Unadjusted* % [95% CI]	*Adjusted** % [95% CI]	*Unadjusted* % [95% CI]	*Adjusted** % [95% CI]
Cleft lip	48 [41, 55]	51 [44, 58]	52 [45, 58]	56 [49, 62]	33 [24, 42]	29 [21, 38]	16 [9, 22]	17 [10, 23]
Spina bifida	64 [54, 73]	65 [56, 73]	66 [58, 75]	70 [62, 77]	30 [19, 40]	27 [17, 37]	30 [21, 39]	28 [19, 36]
CHD	42 [36, 47]	43 [37, 49]	46 [41, 52]	49 [44, 55]	36 [29, 44]	36 [28, 44]	31 [25, 36]	34 [21, 40]
Down syndrome	67 [61, 73]	62 [55, 68]	76 [71, 81]	72 [67, 78]	28 [21, 34]	29 [22, 36]	27 [20, 33]	24 [18, 30]
Down syndrome with CHD	60 [47, 74]	55 [40, 68]	83 [73, 93]	80 [69, 91]	33 [18, 47]	28 [13, 42]	20 [8, 31]	17 [6, 27]

†Total number of participants excluding 'not applicable' responses. Missing data: planned tests/procedures (n=8), appointments rescheduled as virtual (n=8), virtual appointments rated as 'poor' (n=11), problems accessing medication (n=8).

*Adjusted by parental country of residence, age, and education level.

CI = confidence intervals; CHD=congenital heart defects

For peer review only - http://bmjopen.bmj.com/site/about/guidelines.xhtml

DISCUSSION

Main findings

This study provides a snapshot of the healthcare experiences of children with CAs, and their caregivers' experiences of support across Europe, one year into the COVID-19 pandemic. To our knowledge, this is the first multi-national survey exploring this topic. Overall, a high proportion of participants reported disruptions to their child's routine care. Cancellations and postponements of healthcare appointments were highest in the UK and Poland, with two-thirds of participants reporting cancelled/postponed tests and procedures compared with ~20% in Germany and Belgium/Netherlands, and a third of participants reporting cancelled/postponed surgeries compared with only 8% in Germany. Disruptions appeared to have an impact on some children, with close to a third of participants reporting that their child's physical health had been moderately to severely affected by changes to their treatment, which was most apparent in Poland (43%). Around 60% of the sample reported face-to-face appointments being re-scheduled as virtual, a shift also seen in patients with diabetes(27) and obesity.(28) The quality of virtual appointments was rated as 'poor' by a third of participants, which is somewhat higher than in a similar survey of patients with systemic sclerosis.(29) Medical sources of support had the lowest satisfaction ratings across countries, and were particularly low in Poland, the UK and Italy. However, Poland had the highest satisfaction ratings for support from patient organisations and peers.

Few differences were found in outcomes according to CA type, and these were limited to items about the provision of care. Unlike the impact of country, there were no consistent patterns according to the anomaly type, suggesting that the geographical location of participants had more of an influence on healthcare experiences than the child's specific health condition. It was not possible to quantify the depth and duration of COVID-19 containment strategies (which will have influenced the delivery of care) to help explain these cross-country variations in outcomes. This was because the timeframe for the survey started from the beginning of the pandemic to the recruitment period (March-July 2021), and these strategies varied considerably over time, both regionally, within each country, and internationally.

Whilst acknowledging that a range of factors may underpin differences across countries, a possible hypothesis is that these findings are indicative of existing vulnerabilities within local healthcare systems, with lower resourced systems being less able to meet the needs of patients during the pandemic. In relation to the healthcare workforce, recent figures from the Organisation for Economic Co-operation and Development (OECD) indicate that Poland had the lowest numbers of practising doctors per head in Europe (2.3/1,000), closely followed by the UK (2.8/1,000). In

For peer review only - http://bmjopen.bmj.com/site/about/guidelines.xhtml

contrast, Germany had one of the highest numbers per head in Europe (4.4/1,000).(30) Among European countries, Poland, Italy and the UK also had below average numbers of practicing nurses per head, a factor associated with patient satisfaction with care,(31) which ranged from 5.1-7.8/1,000.(30) In comparison, the Netherlands, Belgium, and Germany all had above average figures, 11.1, 11.2 and 13.1/1,000, respectively. A larger number of healthcare workers is likely to have helped with increased demand during the pandemic, and helped mitigate the consequences of staff sickness.(30)

Related to this is the high level of migration of doctors and nurses from Poland.(32) This has resulted in a relatively old healthcare workforce in Poland (24.5% of physicians ≥65 years)(33), compared to other countries surveyed in this study, notably the UK (2.1%) and Germany (6.4%).(30) With the risks from COVID-19 increasing with age, this is of particular importance, as older healthcare professionals may have needed to cease working during the pandemic to protect themselves from infection.

Implications and future research

Our survey findings are important and provide useful insights into the provision of care for children with CAs across Europe during the first year of the pandemic. Findings highlight potential weaknesses of healthcare systems in some countries and suggest that long-term systemic action is required to improve patient experiences and outcomes. The situation appears particularly problematic in the UK and Poland, which may benefit from increased resources to provide for this vulnerable group of patients. Patient organisations and charities provide an invaluable source of knowledge and support to parents and carers of children with CAs, and these should be supported, especially in countries where medical capacity to meet patients' needs may be stretched.

As with many other patient groups, it is clear that the COVID-19 pandemic has had an impact on the experiences of children living with a serious health condition and their families.(17, 18, 34) This particular survey suggests disruptions to care for children, with potential impacts on the child's health. Considering the limitations of this study, it will be important to investigate the impact of the COVID-19 pandemic on the delivery of paediatric services across Europe using population-based data. With the proliferation of telemedicine to deliver care during the pandemic, assessing how effective these virtual strategies have been in ensuring parent satisfaction with care and support from medical professionals will be of great need.

For peer review only - http://bmjopen.bmj.com/site/about/guidelines.xhtml

CONCLUSION

The COVID-19 pandemic continues to put immense pressure on healthcare systems worldwide. At the time of writing, Europe has once again been flagged as the epicentre of the pandemic by the World Health Organisation (WHO)(35). Our survey findings highlight disruptions to the delivery of care across Europe, particularly in the UK and Poland, which raises questions about the ability of the healthcare systems within these countries to meet the needs of children with CAs and their families, and a need for increased resources.

ACKNOWLEDGEMENTS

We thank the following people for their support in developing the survey and supporting its dissemination in Poland: Dominika Madaj-Solberg (Spina Foundation, Katowice, Poland), Tomek i Kasia Grybek (Borys the Hero Foundation, Gdańsk, Poland), Halina Grzymisławska-Słowińska (Fundacja TAK dla Samodzielności, Poznań, Poland), Anna Latos (special educator, Bydgoszcz, Poland), Prof. Jolanta Wierzba (Med.Univ. Gdańsk, Poland), Prof. Robert Śmigiel (Med. Univ. Wrocław, Poland), Prof. Olga Haus (Coll.Med. UMK, Bydgoszcz, Poland), and Dorota Trześniewska (parent, Poznań, Poland). We are very grateful to the following people for translating the survey and supporting the recruitment of participants: Esben Garne Holm, Juan Rico, Dr. Nadia Assanta (Fondazione Toscana Gabriele Monasterio), Dr. Giada Cavazzuti (Associazione "Un cuore, un mondo"), Dr. Elisabetta Lapi, Dr. Antonella Falugiani (Associazione "Trisomia 21 Onlus"), Dr. Alessandro Giacomina, Dr. Marina Rossi (AOU Pisana), Jürgen Wolters (Arbeitsgemeinschaft Spina Bifida und Hydrocephalus, ASBH), Dr Annett Lambrecht (Department of Pediatric Cardiology, University Hospital Magdeburg), Dr Christian Zahl (Department of Oral and Maxillofacial Surgery, University Hospital Magdeburg).

For peer review only

ETHICS APPROVAL

Ethical approval for the study was granted by the St George's (University of London) Research Ethics Committee on 18th December 2020 (reference number: 2020.0311). In Poland, ethical approval was granted on 10th December 2020 by the Bioethics Committee at the Poznań University of Medical Sciences (reference number: 882/20). In Croatia, ethical approval was granted on 10th December 2020 by the Ethics Committee of the Children's Hospital Zagreb (Protocol No: 02-23/43-1-20 Zagreb). In Spain, ethical approval was granted on 21st December 2020 by the Clinical Investigation Ethics Committee of the "Dirección General de Salud Pública y Centro Superior de Investigación en Salud Pública" (reference number: 20201221/05). In Belgium, ethical approval was granted on 1st March 2021 by the Ethics Committee of the University Hospital of Antwerp (reference: 21/06/084). In

For peer review only - http://bmjopen.bmj.com/site/about/guidelines.xhtml

Portugal, ethical approval was granted on 16th March by the Ethics Committee of the National Institute of Health Doutor Ricardo Jorge (CES-INSA). In Germany, ethical approval was granted on 15th April 2021 by the Medical Faculty of the Otto-von-Guericke-University Magdeburg Research Ethics Committee (reference number: 44/21). In Italy, ethical approval was granted on 14th June 2021 by the Research Ethics and Integrity Committee of the National Research Council Institute of Clinical Physiology in Pisa (CNR-INF) (protocol number 0065527/2019). No local ethical approvals were required in Denmark (Lillebaelt Hospital – University Hospital of Southern Denmark) or the Netherlands (University Medical Center Groningen).

DATA AVAILABILITY STATEMENT

The datasets analysed during the current study are available from the corresponding author on reasonable request.

CONTRIBUTERS

ALB conceptualised the study. ALB, EM, and JKM contributed to the study design. EM led the survey development, translation, and recruitment of participants. EM and JKM conducted the data analysis. ALB, JKM, and JR critically revised the manuscript. AJD, IB, CCC, EDH, EG, EM, LG, AJS, RT, CMD, CNP, AJN, AN, LO, LPR, AP, and AR oversaw the translation of the survey and recruited participants. EM drafted the manuscript. All authors contributed to, read and approved the final manuscript.

FUNDING

This project has received funding from the European Union's Horizon 2020 research and innovation programme under grant agreement No 733001. Start date: 1 Jan 2017. Duration: 5 years and 5 months. The views presented here are those of the authors only, and the European Commission is not responsible for any use that may be made of the information presented here.

COMPETING INTERESTS

All authors have completed the ICMJE uniform disclosure form at http://www.icmje.org/disclosure-of-interest/ and declare: all authors had financial support from the European Union's Horizon 2020 research and innovation programme for the submitted work; no financial relationships with any organisations that might have an interest in the submitted work in the previous three years; no other relationships or activities that could appear to have influenced the submitted work.

For peer review only - http://bmjopen.bmj.com/site/about/guidelines.xhtml

TRANSPARENCY DECLARATION

The corresponding author affirms that the manuscript is an honest, accurate, and transparent account of the study being reported; that no important aspects of the study have been omitted; and that any discrepancies from the study as planned (and, if relevant, registered) have been explained.

DISSEMINATION TO STUDY PARTICIPANTS AND RELATED PATIENT AND PATIENT COMMUNITIES

Findings from the study will be shared with members of the public, parents and carers, healthcare professionals and relevant stakeholders via scientific publications, lay reports, social media, and conferences.

For peer review only

REFERENCES

1. Smolic S, Cipin I, Medimurec P. Access to healthcare for people aged 50+ in Europe during the COVID-19 outbreak. European Journal of Ageing. 2021:1-17.
2. Cena L, Rota M, Calza S, Massardi B, Trainini A, Stefana A. Estimating the Impact of the COVID-19 Pandemic on Maternal and Perinatal Health Care Services in Italy: Results of a Self-Administered Survey. Frontiers in Public Health. 2021;9:701638.
3. van Veenendaal NR, Deierl A, Bacchini F, O'Brien K, Franck LS, & the International Steering Committee for Family Integrated Care. Supporting parents as essential care partners in neonatal units during the SARS-CoV-2 pandemic. Acta Paediatr. 2021;110:2008-22.
4. Gardner T, Fraser C. Elective care: how has COVID-19 affected the waiting list? London: The Health Foundation. 2021:Available from: https://bit.ly/3EKqvRL [accessed 1 Nov 2021].
5. Chiumento A, Baines P, Redhead C, Fovargue S, Draper H, Frith L. Which ethical values underpin England's National Health Service reset of paediatric and maternity services following COVID-19: a rapid review. BMJ Open. 2021;11:e049214.
6. Colvin L, Bower C. A retrospective population-based study of childhood hospital admissions with record linkage to a birth defects registry. BMC Pediatr. 2009;9:32.
7. Rosano A, Botto LD, Botting B, Mastroiacovo P. Infant mortality and congenital anomalies from 1950 to 1994: an international perspective. Journal of Epidemiology and Community Health. 2000;54(9):660-6.
8. Department of Health. National framework for children and young peoples continuing care. London: Department of Health; 2016.
9. Razzaghi H, Dawson A, Grosse SD, Allori AC, Kirby RS, Olney RS, et al. Factors associated with high hospital resource use in a population-based study of children with orofacial clefts. Birth Defects Res A Clin Mol Teratol. 2015;103(2):127-43.
10. Fitzgerald P, Leonard H, Pikora TJ, Bourke J, Hammond G. Hospital admissions in children with down syndrome: experience of a population-based cohort followed from birth. PLoS ONE. 2013;8(8):e70401.
11. Bishop CF, Small N, Parslow R, Kelly B. Healthcare use for children with complex needs: using routine health data linked to a multiethnic, ongoing birth cohort. BMJ Open. 2018;8(3):e018419.
12. Ludvigsson JF. Systematic review of COVID-19 in children shows milder cases and a better prognosis than adults. Acta Paediatr. 2020;109(6):1088-95.
13. Malle L, Gao C, Hur C, Truong HQ, Bouvier NM, Percha B, et al. Individuals with Down syndrome hospitalized with COVID-19 have more severe disease. Genet Med. 2021;23(3):576-80.
14. Clift AK, Coupland CAC, Keogh RH, Hemingway H, Hippisley-Cox J. COVID-19 Mortality Risk in Down Syndrome: Results From a Cohort Study of 8 Million Adults. Ann Intern Med. 2021;174(4):572-6.
15. Malviya A, Yadav R. COVID -19 pandemic and paediatric population with special reference to congenital heart disease. Indian Heart J. 2020;72(3):141-4.
16. The Cleft Lip and Palate Association. Summer Survey 2020: The Results. Available from: https://www.clapa.com/news-item/summer-survey-2020-the-results/ [accessed 2 Nov 2021]. 2020.
17. Wray J, Pagel C, Chester AH, Kennedy F, Crowe S. What was the impact of the first wave of COVID-19 on the delivery of care to children and adults with congenital heart disease? A qualitative study using online forums. BMJ Open. 2021;11:e049006.
18. Marino LV, Wagland R, Culliford DJ, Bharucha T, Sodergren SC, Darlington AE. "No Official Help Is Available"-Experience of Parents and Children With Congenital Heart Disease During COVID-19. World Journal for Pediatric and Congenital Heart Surgery. 2021;12(4):500-7.
19. Cuschieri S. The STROBE guidelines. Saudi J Anaesth. 2019;13(Suppl 1):S31-S4.
20. Kuliś D, Bottomley A, Velikova G, Greimel E, Koller M, group ObotEOfRaToCEQoL. EORTC Quality of Life Group Translation Procedure (4th edition). Available from: https://www.eortc.org/app/uploads/sites/2/2018/02/translation_manual_2017.pdf [accessed 14 Nov 2021]. 2017.

For peer review only - http://bmjopen.bmj.com/site/about/guidelines.xhtml

21. Harris PA, Taylor R, Thielke R, Payne J, Gonzalez N, Conde JG. Research electronic data capture (REDCap)--a metadata-driven methodology and workflow process for providing translational research informatics support. J Biomed Inform. 2009;42(2):377-81.
22. StataCorp. Stata Statistical Software: Release 17. College Station, TX: StataCorp LLC2021.
23. Leirgul E, Fomina T, Brodwall K, al e. Birth prevalence of congenital heart defects in Norway 1994–2009 - a nationwide study. Am Heart J. 2014;168(6):956-64.
24. McRobert CJ, Hill JC, Smale T, Hay EM, van der Windt DA. A multi-modal recruitment strategy using social media and internet-mediated methods to recruit a multidisciplinary, international sample of clinicians to an online research study. PLoS ONE. 2018;13(7):e0200184.
25. Liu M, Wronski L. Examining completion rates in web surveys via over 25,000 real-world surveys. Social Science Computer Review. 2018;36:116-24.
26. Bosnjak M, Tuten TL. Classifying Response Behaviors in Web-based Surveys. Journal of Computer-Mediated Communication. 2001;6(3).
27. Forde R, Arente L, Ausili D, De Backer K, Due-Christensen M, Epps A, et al. The impact of the COVID-19 pandemic on people with diabetes and diabetes services: A pan-European survey of diabetes specialist nurses undertaken by the Foundation of European Nurses in Diabetes survey consortium. Diabet Med. 2021;38(5):e14498.
28. Dicker D, Bettini S, Farpour-Lambert N, Fruhbeck G, Golan R, Goossens G, et al. Obesity and COVID-19: The Two Sides of the Coin. Obesity Facts. 2020;13(4):430-8.
29. Hughes M, Pauling JD, Moore A, Jones J. Impact of Covid-19 on clinical care and lived experience of systemic sclerosis: An international survey from EURORDIS-Rare Diseases Europe. Journal of Scleroderma and Related Disorders. 2021;6(2):133-8.
30. OECD, European Union. Health at a Glance: Europe 2020. State of Health in the EU Cycle. OECD Publishing; Paris. Available from: https://doi.org/10.1787/82129230-en [accessed 26 Nov 2021]. 2020.
31. Aiken LH, Sloane DM, Ball J, Bruyneel L, Rafferty AM, Griffiths P. Patient satisfaction with hospital care and nurses in England: an observational study. BMJ Open. 2018;8(1):e019189.
32. Szpakowski R, Dykowska G, Fronczak A, Zajac P, Czerw A. Migrations of nurses and doctors from Poland: data for the years 2014-2020 based on the sample of the capital city of Warsawgamma. Archives of Medical Science. 2019;15(3):811-20.
33. Polish Ministry of Health. Biuletyn Statystyczny 2020 - Centrum e-Zdrowia. Available from: https://www.cez.gov.pl/projekty/statystyka/biuletyn-statystyczny/ [accessed 29 Nov 2021]. 2020.
34. Darlington A, Morgan J, Wagland R, Sodergren S, Culliford D, Gamble A, et al. COVID-19 and children with cancer: Parents' experiences, anxieties and support needs. Pediatr Blood Cancer. 2021;68:e28790.
35. WHO. Statement – Update on COVID-19: Europe and central Asia again at the epicentre of the pandemic. Available from: https://www.euro.who.int/en/media-centre/sections/statements/2021/statement-update-on-covid-19-europe-and-central-asia-again-at-the-epicentre-of-the-pandemic [accessed 26 Nov 2021]. 2021.

For peer review only - http://bmjopen.bmj.com/site/about/guidelines.xhtml

FIGURE LEGEND

Figure 1 Proportion* of participants reporting 'cancelled or postponed' routine appointments, planned tests or procedures, and planned surgeries with 95% confidence intervals, by country.

*Adjusted by congenital anomaly type, parental age, and education level.

Figure 2 Proportion* of participants reporting that their child's health had been 'moderately to severely' compromised following changes to their child's treatment with 95% confidence intervals, by country.

*Adjusted by congenital anomaly type, parental age, and education level.

Figure 3 Proportion* of participants reporting that their child's physical health was 'worse', 'about the same' or 'better' than it was prior to the pandemic with 95% confidence intervals, by country.

*Adjusted by congenital anomaly type, parental age, and education level.

Figure 4 Proportion* of participants reporting they would have liked more support during the pandemic 'very much' with 95% confidence intervals, by country.

*Adjusted by congenital anomaly type, parental age, and education level.

For peer review only

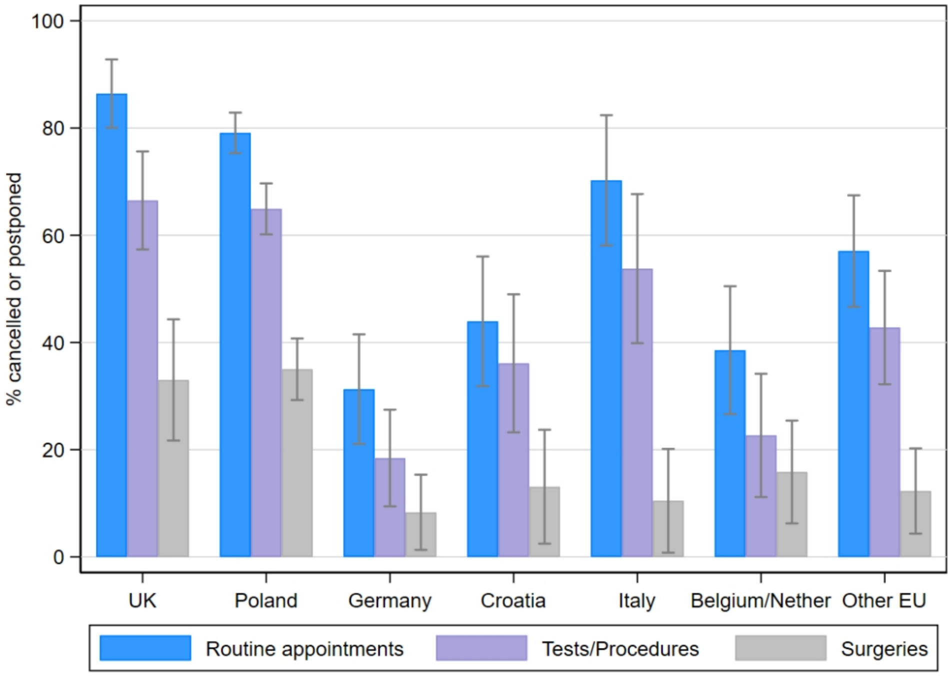

415x301mm (72 x 72 DPI)

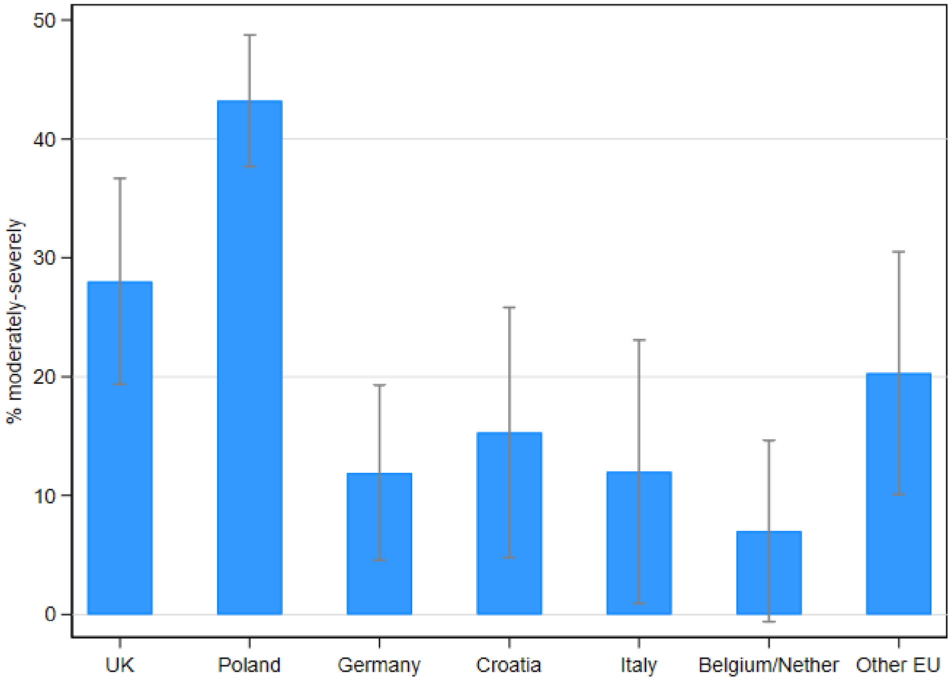

242x176mm (72 x 72 DPI)

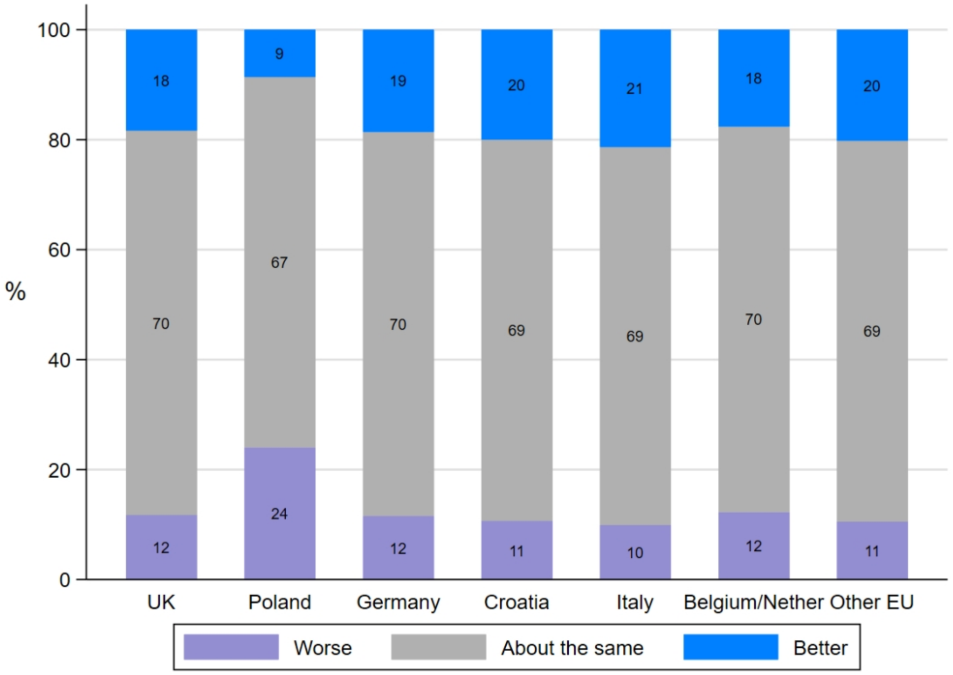

415x301mm (72 x 72 DPI)

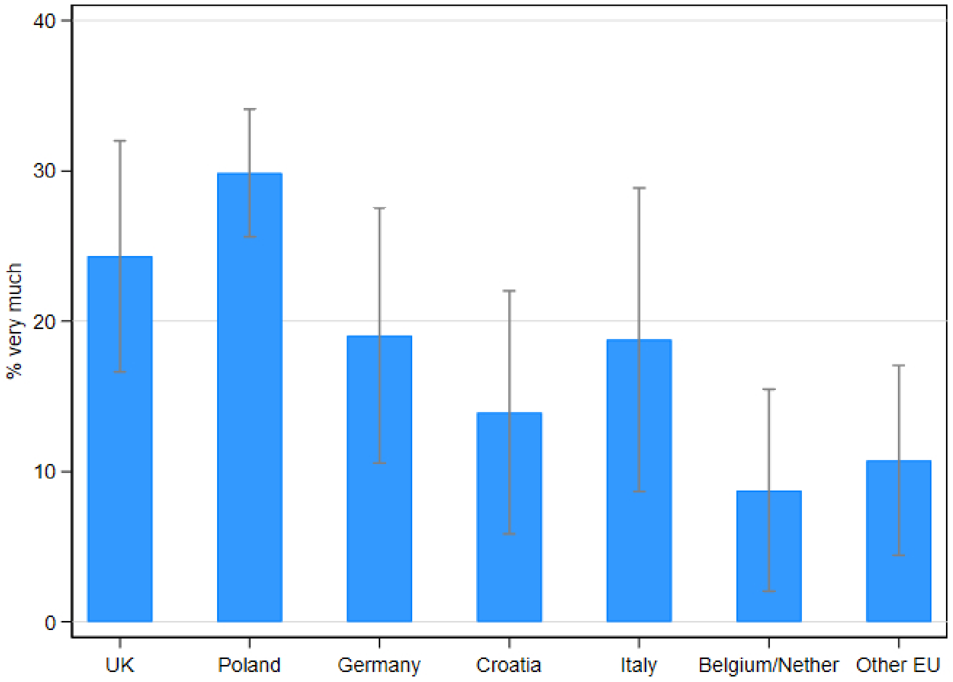

242x176mm (72 x 72 DPI)

STROBE Statement—Checklist of items that should be included in reports of ***cross-sectional studies***

	Item No	Recommendation	Page No
**Title and abstract**	1	(*a*) Indicate the study's design with a commonly used term in the title or the abstract	1, 3
		(*b*) Provide in the abstract an informative and balanced summary of what was done and what was found	3
**Introduction**			
Background/rationale	2	Explain the scientific background and rationale for the investigation being reported	4
Objectives	3	State specific objectives, including any prespecified hypotheses	4
**Methods**			
Study design	4	Present key elements of study design early in the paper	5, 6
Setting	5	Describe the setting, locations, and relevant dates, including periods of recruitment, exposure, follow-up, and data collection	5-7
Participants	6	(*a*) Give the eligibility criteria, and the sources and methods of selection of participants	5
Variables	7	Clearly define all outcomes, exposures, predictors, potential confounders, and effect modifiers. Give diagnostic criteria, if applicable	6
Data sources/ measurement	8*	For each variable of interest, give sources of data and details of methods of assessment (measurement). Describe comparability of assessment methods if there is more than one group	6
Bias	9	Describe any efforts to address potential sources of bias	7
Study size	10	Explain how the study size was arrived at	8
Quantitative variables	11	Explain how quantitative variables were handled in the analyses. If applicable, describe which groupings were chosen and why	7-8
Statistical methods	12	(*a*) Describe all statistical methods, including those used to control for confounding	7-8
		(*b*) Describe any methods used to examine subgroups and interactions	8
		(*c*) Explain how missing data were addressed	8
		(*d*) If applicable, describe analytical methods taking account of sampling strategy	n/a
		(*e*) Describe any sensitivity analyses	n/a
**Results**			
Participants	13*	(a) Report numbers of individuals at each stage of study—eg numbers potentially eligible, examined for eligibility, confirmed eligible, included in the study, completing follow-up, and analysed	8
		(b) Give reasons for non-participation at each stage	n/a
		(c) Consider use of a flow diagram	n/a
Descriptive data	14*	(a) Give characteristics of study participants (eg demographic, clinical, social) and information on exposures and potential confounders	8-9
		(b) Indicate number of participants with missing data for each variable of interest	8
Outcome data	15*	Report numbers of outcome events or summary measures	8
Main results	16	(*a*) Give unadjusted estimates and, if applicable, confounder-adjusted estimates and their precision (eg, 95% confidence interval). Make clear which confounders were adjusted for and why they were included	7 (in tables)

For peer review only - http://bmjopen.bmj.com/site/about/guidelines.xhtml

For peer review only

		(*b*) Report category boundaries when continuous variables were categorized	n/a
		(*c*) If relevant, consider translating estimates of relative risk into absolute risk for a meaningful time period	n/a
Other analyses	17	Report other analyses done—eg analyses of subgroups and interactions, and sensitivity analyses	n/a
**Discussion**			
Key results	18	Summarise key results with reference to study objectives	11
Limitations	19	Discuss limitations of the study, taking into account sources of potential bias or imprecision. Discuss both direction and magnitude of any potential bias	13
Interpretation	20	Give a cautious overall interpretation of results considering objectives, limitations, multiplicity of analyses, results from similar studies, and other relevant evidence	12
Generalisability	21	Discuss the generalisability (external validity) of the study results	13
**Other information**			
Funding	22	Give the source of funding and the role of the funders for the present study and, if applicable, for the original study on which the present article is based	17

*Give information separately for exposed and unexposed groups.

Note: An Explanation and Elaboration article discusses each checklist item and gives methodological background and published examples of transparent reporting. The STROBE checklist is best used in conjunction with this article (freely available on the Web sites of PLoS Medicine at http://www.plosmedicine.org/, Annals of Internal Medicine at http://www.annals.org/, and Epidemiology at http://www.epidem.com/). Information on the STROBE Initiative is available at www.strobe-statement.org.

For peer review only

BMJ Open

COVID-19 and children with congenital anomalies: a European survey of parents' experiences of healthcare services.

Journal:	*BMJ Open*
Manuscript ID	bmjopen-2022-061428.R1
Article Type:	Original research
Date Submitted by the Author:	05-May-2022
Complete List of Authors:	Latos-Bieleńska, Anna; University of Medical Sciences, Department of Medical Genetics<br>Marcus, Elena; St George's University of London, Population Population Health Research Institute<br>Jamry-Dziurla, Anna; University of Medical Sciences, Department of Medical Genetics<br>Rankin, Judith; Newcastle University, Population Health Sciences Institute<br>Barisic, Ingeborg; Children's University Hospital of Zagreb, Centre of Excellence for Reproductive and Regenerative Medicine<br>Cavero- Carbonell, Clara; Fundacio per al Foment de la Investigacio Sanitaria i Biomedica, Rare diseases research unit<br>Den Hond, Elly; Provincial Institute for Hygiene<br>Garne, Ester; Hospital Lillebaelt, Kolding, Paediatric Department<br>Genard, Lucas; Provincial Institute for Hygiene<br>Santos, Ana ; National Health Institute Doutor Ricardo Jorge Department of Epidemiology<br>Lutke, Renee; University Medical Center Groningen, Department of Genetics<br>Dias, Carlos; National Institute of Health, Department of Epidemiology<br>Neergaard Pedersen, Christina; Hospital Lillebaelt, Kolding, Paediatric Department<br>Neville, Amanda; University de Ferrara, IMER Registry (Emilia Romagna Registry of Birth Defects)<br>Niemann, Annika; Medical Faculty Otto-von-Guericke University, Malformation Monitoring Centre Saxony-Anhalt<br>Odak, Ljubica; Children's Hospital Zagreb, Centre of Excellence for Reproductive and Regenerative Medicine, Medical School University of Zagreb<br>Páramo-Rodríguez, Lucía; Fundacio per al Foment de la Investigacio Sanitaria i Biomedica, Rare diseases research unit<br>Pierini, Anna; Institute of Clinical Physiology, Unit of Epidemiology of Rare Diseases and Congenital Anomalies<br>Rissmann, Anke; Medical Faculty Otto-von-Guericke University, Malformation Monitoring Centre Saxony-Anhalt<br>Morris, Joan; St George's, University of London, Population Health Research Institute
Primary Subject	Paediatrics

Heading:	
Secondary Subject Heading:	Health services research
Keywords:	COVID-19, PAEDIATRICS, International health services < HEALTH SERVICES ADMINISTRATION & MANAGEMENT

SCHOLARONE™
Manuscripts

I, the Submitting Author has the right to grant and does grant on behalf of all authors of the Work (as defined in the below author licence), an exclusive licence and/or a non-exclusive licence for contributions from authors who are: i) UK Crown employees; ii) where BMJ has agreed a CC-BY licence shall apply, and/or iii) in accordance with the terms applicable for US Federal Government officers or employees acting as part of their official duties; on a worldwide, perpetual, irrevocable, royalty-free basis to BMJ Publishing Group Ltd ("BMJ") its licensees and where the relevant Journal is co-owned by BMJ to the co-owners of the Journal, to publish the Work in this journal and any other BMJ products and to exploit all rights, as set out in our licence.

The Submitting Author accepts and understands that any supply made under these terms is made by BMJ to the Submitting Author unless you are acting as an employee on behalf of your employer or a postgraduate student of an affiliated institution which is paying any applicable article publishing charge ("APC") for Open Access articles. Where the Submitting Author wishes to make the Work available on an Open Access basis (and intends to pay the relevant APC), the terms of reuse of such Open Access shall be governed by a Creative Commons licence – details of these licences and which Creative Commons licence will apply to this Work are set out in our licence referred to above.

Other than as permitted in any relevant BMJ Author's Self Archiving Policies, I confirm this Work has not been accepted for publication elsewhere, is not being considered for publication elsewhere and does not duplicate material already published. I confirm all authors consent to publication of this Work and authorise the granting of this licence.

For peer review only

COVID-19 and children with congenital anomalies: a European survey of parents' experiences of healthcare services.

Anna Latos-Bielenska[1]*, Elena Marcus[2]*, Anna Jamry-Dziurla[1], Judith Rankin[3], Ingeborg Barišić[4], Clara Cavero-Carbonell[5], Elly Den Hond[6], Ester Garne[7], Lucas Genard[6], Ana João Santos[8], L Renée Lutke[9], Carlos Matias Dias[8], Christina Neergaard Pedersen[7], Amanda J Neville[10], Annika Niemann[11], Ljubica Odak[4], Lucía Páramo-Rodríguez[5], Anna Pierini[12], Anke Rissmann[11], Joan K Morris[2].

[1]Chair and Department of Medical Genetics, Poznan University of Medical Sciences, Collegium Maius, Fredry 10, 61-701, Poznań, Poland.

Anna Latos-Bielenska, professor.

Anna Jamry-Dziurla, deputy registry co-ordinator.

[2]Population Health Research Institute, St George's, University of London, Cranmer Terrace, London SW17 0RE, United Kingdom.

Elena Marcus, postdoctoral researcher.

Joan K Morris, professor of statistics.

[3]Population Health Sciences Institute, Newcastle University, Newcastle upon Tyne, NE1 7RU, United Kingdom.

Judith Rankin, professor of maternal and child health.

[4]Children's Hospital Zagreb, Centre of Excellence for Reproductive and Regenerative Medicine, Medical School University of Zagreb, Ul. Vjekoslava Klaića 16, 10000, Zagreb, Croatia.

Ingeborg Barišić, professor.

Ljubica Odak, consultant paediatrician.

[5]Rare Diseases Research Unit, Foundation for the Promotion of Health and Biomedical Research in the Valencian Region, Av. de Catalunya, 21, 46020 València, Spain.

Clara Cavero-Carbonell, researcher and head of the Rare Diseases Research Unit.

Lucía Páramo-Rodríguez, research assistant.

[6]Provincial Institute for Hygiene (PIH), Kronenburgstraat 45, 2000 Antwerpen, Belgium.

Elly Den Hond, senior research associate.

For peer review only - http://bmjopen.bmj.com/site/about/guidelines.xhtml

Lucas Genard, researcher.

[7]University Hospital Lillebaelt, Sygehusvej 24, 6000 Kolding, Denmark.

Ester Garne, consultant pediatrician and associate professor.

Christina Neergaard Pedersen, specialty registrar.

[8]Department of Epidemiology, National Institute of Health Doctor Ricardo Jorge, Av. Padre Cruz, 1600-609 Lisboa, Portugal.

Ana João Santos, senior technician.

Carlos Matias Dias, department co-ordinator.

[9]Department of Genetics, University Medical Center, University of Groningen, 9712 CP Groningen, Netherlands.

L Renée Lutke, pharmacist/researcher

[10] IMER Registry (Emilia Romagna Registry of Birth Defects), Center for Clinical and Epidemiological Research, University of Ferrara, Azienda Ospedaliero- Universitaria di Ferrara, Corso Giovecca, 203, 44121 Ferrara (Italy).

Amanda J Neville, EUROCAT registry lead.

[11]Malformation Monitoring Centre Saxony-Anhalt, Medical Faculty, Otto-von-Guericke-University Magdeburg, Leipziger Str. 44, 39120 Magdeburg, Germany.

Anke Rissmann, consultant paediatrician and registry leader.

Annika Niemann, research associate.

[12]Unit of Epidemiology of Rare Diseases and Congenital Anomalies, Institute of Clinical Physiology, National Research Council, Via Giuseppe Moruzzi, 1, 56124 Pisa, Italy.

Anna Pierini, senior researcher.

***Joint first author: these authors contributed equally**

Corresponding author: Dr Elena Marcus, Population Health Research Institute, St George's, University of London, Cranmer Terrace, London SW17 0RE, UK. Email: emarcus@sgul.ac.uk.

Word Count: 4,421

For peer review only - http://bmjopen.bmj.com/site/about/guidelines.xhtml

ABSTRACT

Objective

To survey parents and carers of children with a congenital anomaly (CA) across Europe about their experiences of healthcare services and support during the COVID-19 pandemic.

Design

Cross-sectional study.

Setting

Online survey in 10 European countries, open from 8th March 2021 to 14th July 2021.

Population

1,070 parents and carers of children aged 0-10 years with a cleft lip, spina bifida, congenital heart defect (CHD) requiring surgery, and/or Down syndrome.

Main outcome measures

Parental views about: the provision of care for their child (cancellation/postponement of appointments, virtual appointments, access to medication), the impact of disruptions to healthcare on their child's health and well-being, and satisfaction with support from medical sources, organisations and close relationships.

Results

Disruptions to healthcare appointments were significantly higher (p<0.001) in the UK and Poland, with approximately two-thirds of participants reporting 'cancelled or postponed' tests (67/101; 256/389) and procedures compared with approximately 20% in Germany (13/74) and Belgium/Netherlands (11/55). A third of participants in the UK and Poland reported 'cancelled or postponed' surgeries (22/72; 98/266) compared with only 8% in Germany (5/64). In Poland, 43% (136/314) of parents reported that changes to their child's ongoing treatment had moderately to severely affected their child's health, significantly higher than all other countries (p<0.001). Satisfaction ratings for support from general practitioners were lowest in the UK and Poland, and lowest in Poland and Italy for specialist doctors and nurses.

Conclusion

A large proportion of participants reported disruptions to healthcare during the pandemic, which for some had a significant impact on their child's health. Regional differences in disruptions raise questions about the competence of certain healthcare systems to meet the needs of this vulnerable group of patients and indicate improvements should be strived for in some regions.

Keywords

congenital anomaly, COVID-19, child, parental experience, provision of healthcare, support, survey

For peer review only - http://bmjopen.bmj.com/site/about/guidelines.xhtml

STRENGTHS AND LIMITATIONS

- Surveys the experiences of a large total number of parents and carers across several European countries and congenital anomaly types. The proportion of each CA type in the study sample reflects the relative number of live births with each CA in Europe.
- High item-level response rates, suggests that survey items were relevant to participants and easy to complete.
- Potential bias in responses due to the use of social media for recruitment, for example, excluding people living with 'digital poverty' and those who do not engage with patient and parent organisations, limiting the generalisability of findings.
- Inability to conduct a full pilot of the final survey to explore item acceptability, comprehension, and relevance. Possible that there may be some issues with the wording or content of items.

BACKGROUND

The coronavirus disease 2019 (COVID-19) pandemic put pressure on healthcare systems worldwide, causing severe disruptions to the delivery of non-essential services, as staff were re-deployed to acute care, and outpatient treatment and follow-up was reduced due to concerns about viral transmission in hospital.[1-3] Non-urgent elective care was the most heavily impacted, with a record backlog of 5.6 million cases reported in England in July 2021.[4,5]

Congenital anomalies (CAs) are a range of conditions that are present from birth and remain a leading cause of childhood morbidity and long-term disability.[6,7] Children with CAs require regular clinical follow-up,[8] including more frequent primary care appointments, hospital admissions, and surgeries than children without CAs.[9-12] Although children are less affected by SARS-CoV-2 infection than adults,[13] Down syndrome has been indicated as a risk factor of severe disease and mortality,[14,15] and children with underlying conditions may be at increased risk of infection.[16] It is crucial to document the healthcare experiences of children with CAs during this period of increased pressure on healthcare systems, especially as they represent a vulnerable population. Existing research, conducted during the first wave of the pandemic in 2020, suggests a high proportion of cancellations and postponements to pediatric healthcare appointments and treatments in the United States[17] and in Europe.[18-22] Disruptions to the healthcare services of children with CAs were found to cause anxiety for parents,[20] and fear that their child's health may be negatively affected.[22] Corcerns about SARS-CoV-2 infection were also common among parents,[23,24] which coupled with reductions in other communicable infections during the pandemic,[25,26] resulted in fewer visits to clinics[25,27] and

For peer review only - http://bmjopen.bmj.com/site/about/guidelines.xhtml

emergency departments[26,28] in 2020. Parents reported a lack of support from healthcare professionals, including the absence of specific COVID-19 related guidance for children.[22,29]

This paper describes a cross-sectional online survey which explored the views of parents and carers of children with CAs about: (a) their healthcare experiences, and (b) their experiences of support, one year into the pandemic. The survey was conducted as part of a collaborative European project, “Establishing a linked European Cohort of Children with CAs (EUROlinkCAT)”,[30] which aims to investigate health and educational outcomes in children born with CAs using population-based data. Due to differences in the level of restrictions, healthcare systems, and the availability of resources between countries, the survey was conducted in several European countries, to explore possible variations in the provision of care.

METHODS

This study is reported following the Strengthening the Reporting of Observational studies in Epidemiology (STROBE) guidelines.[31] The findings presented are a subsection of a cross-sectional online survey, conducted by the EUROlinkCAT team, which explored the wider information and support needs of parents and carers of children with CAs in 10 European countries. This paper focuses on the healthcare experiences and health status of children during the COVID-19 pandemic, and parent and carer experiences of support. The survey was launched in the United Kingdom (UK) and Poland on 8th March 2021 and kept open until 14th July 2021. Ethics approval for the study was granted by the St George’s (University of London) Research Ethics Committee on 18th December 2020 (reference number: 2020.0311). Local ethics approvals (or evidence that no further approvals were required) were obtained from each participating country. The survey was launched in a staggered manner in each country, as and when translations were finalised and approvals granted (Table 1).

For peer review only - http://bmjopen.bmj.com/site/about/guidelines.xhtml

Table 1 Recruitment period and participant characteristics by country group.

Characteristic	All	UK	Poland	Germany	Croatia	Italy	Belgium/ Netherlands	Other EU†
**Recruitment period¥**								
Start date	-	8 Mar 2021	8 Mar 2021	11 May 2021	26 Apr 2021	16 Jun 2021	19 Apr 2021	6 Apr 2021
End date	-	14 Jul 2021	14 Jul 2021	14 Jul 2021	14 Jul 2021	31 Jul 2021	14 Jul 2021	14 Jul 2021
**N**	986	120	476	97	68	59	74	92
**Age**								
≤30	162 (17%)	18 (15%)	93 (20%)	13 (13%)	8 (12%)	4 (7%)	15 (20%)	11 (12%)
31-40	516 (53%)	53 (45%)	264 (56%)	51 (53%)	37 (55%)	27 (46%)	35 (47%)	49 (53%)
>40	301 (31%)	47 (40%)	115 (24%)	33 (34%)	22 (33%)	28 (47%)	24 (32%)	34 (35%)
**Relation to child**								
Mother	911 (92%)	116 (97%)	449 (94%)	81 (84%)	63 (93%)	52 (88%)	64 (86%)	86 (95%)
Father	65 (7%)	2 (2%)	24 (5%)	13 (13%)	5 (7%)	6 (10%)	10 (14%)	5 (5%)
Other‡	8 (1%)	1 (1%)	3 (1%)	3 (3%)	-	1 (2%)	-	-
**Employment**								
Employed	586 (60%)	81 (68%)	223 (47%)	61 (62%)	54 (79%)	44 (75%)	61 (82%)	62 (69%)
Homemaker/carer	301 (31%)	36 (30%)	198 (42%)	27 (29%)	7 (10%)	11 (19%)	8 (11%)	14 (16%)
Other*	94 (9%)	3 (3%)	52 (11%)	9 (9%)	7 (10%)	4 (7%)	5 (7%)	14 (16%)
**Education**								
School ≤18 years	390 (40%)	44 (37%)	163 (35%)	61 (67%)	19 (28%)	30 (52%)	44 (60%)	29 (32%)
University	482 (49%)	50 (42%)	257 (53%)	27 (29%)	45 (66%)	19 (33%)	29 (39%)	55 (60%)
Post-graduate	106 (11%)	25 (21%)	56 (11%)	3 (3%)	4 (6%)	9 (16%)	1 (1%)	8 (9%)
**Migrant status**								
>10 years/from birth	924 (94%)	111 (93%)	467 (98%)	86 (88%)	64 (94%)	50 (86%)	71 (96%)	75 (81%)
6-10 years	30 (3%)	5 (4%)	5 (1%)	6 (7%)	2 (3%)	4 (7%)	1 (1%)	7 (8%)
1-5 years	28 (3%)	4 (3%)	2 (0.4%)	5 (5%	2 (3%)	4 (7%)	2 (3%)	9 (10%)
<1 year	2 (0.2%)	-	1 (0.2%)	-	-	-	-	1 (1%)

¥The recall period for the survey items was from January 2020 until the time at which participants were recruited.
†Other European countries: Denmark (n=39), Portugal (n=23), Spain (n=16), Ireland (n=5), Bulgaria (n=2), Albania (n=1), Cyprus (n=1), Lithuania (n=1), Norway (n=1), Romania (n=1), Sweden (n=1), Ukraine (n=1).
‡Other family member (n=3), legal guardian related to the child (n=2), legal guardian unrelated to the child (n=3).
*Unemployed (n=56), long-term sick/disabled (n=17), on furlough (n=12), student (n=8), retired (n=1)

For peer review only - http://bmjopen.bmj.com/site/about/guidelines.xhtml

Participants

The survey was open to parents, carers, and guardians (termed henceforth as *parents*) of children up to 10 years of age who have one or more of the following CAs: cleft lip (with or without a cleft palate), spina bifida, CHD which required surgery, and Down syndrome. Due to the high level of heterogeneity across all CAs, these groups were pre-defined and selected to cover different types of impairments, with likely differing impacts on the experiences of the child and parent: (a) physical disability (spina bifida), (b) learning disability (Down syndrome), (c) visible defects (cleft lip), and (d) non-visible defects (CHD). Participants were actively recruited in 10 European countries: Belgium, Croatia, Denmark, Germany, Italy, Netherlands, Poland, Portugal, Spain, and the UK.

Recruitment

Participants were recruited with convenience sampling which was conducted online via social media (Twitter and Facebook), charities and patient organisations within each participating country (e.g. the Down Syndrome Association in the UK), and closed support groups on Facebook. Potential participants were provided with a link to the survey website which included all language versions of the survey. Participants were provided with the participant information sheet at the start of the survey, and depending on local ethics requirements, participants were either required to complete an online consent form or consent was implied by completion of the survey. As the survey was shared across online platforms and by a number of international organisations (e.g. Down Syndrome International), responses were also received from parents living in other European countries (e.g. Ireland), and these were retained in the analysis.

Survey

The content of the survey was developed following a literature review, and input from expert clinicians, parents and educators, academics with expertise in CA research and questionnaire development, and a Public Involvement and Community Engagement (PICE) lead. The survey included the following sections: (1) Parent Demographics (9 items), (2) Child Demographics and Medical Information (7 items), (3) Provision of Healthcare (7 items), (4) Impact on the Child (3 items), and (5) Support for Parents (2 items) (see Supplementary file). Response options varied and comprised: Yes-No (Provision of Healthcare), Not at all-A little-Quite a bit-Very much (Provision of Healthcare), Not at all satisfied-A little satisfied-Quite satisfied-Very satisfied (Support for Parents), and Much worse-Somewhat worse-About the same-Somewhat better-Much better (Impact on Child). All items were close-ended, therefore quantitative data were collected only. In relation to the

For peer review only - http://bmjopen.bmj.com/site/about/guidelines.xhtml

timeframe, participants were asked to reflect on their experiences from the start of the pandemic in January 2020 to the time at which they completed the survey (March-July 2021).

Translation

The survey was developed in English and translated into eight European languages following existing guidance.[32] The Dutch version was used in Belgium and the Netherlands. The survey was initially translated into Polish and Italian to check for any translatability issues, and relevant amendments were subsequently made to the English version accordingly. These languages were selected because they have different origins (Slavic and Romance) with differing translation issues, and the research team included native Polish (ALB, AJ) and native English-Italian bilingual (EM) speakers. Translations were carried out in four steps for each language version: (1) a native speaker of the target language with good command of English conducted the initial translation, (2) the translation was checked by at least one other native speaker of the target language and any problems discussed and reconciled, (3) the survey was back-translated by a native English speaker (or person with a good command of the English language), and who was naïve to the original version, (4) the back-translated survey was reviewed by EM against the original English language version and any semantic or conceptual discrepancies in the back-translation were flagged and discussed with the translators until they were resolved. Due to differences in education systems across Europe, equivalent terminology for participants' education level was not available, and categories were selected to reflect local education systems within each country.

Data collection

Study data were collected and managed using Research Electronic Data Capture[33] (REDCap) tools hosted at St George's, University of London. All data collected were anonymous and it was therefore not possible to verify CA diagnoses. To keep the survey fully anonymous no internet protocol (IP) addresses were collected, so it was not possible to prevent multiple participation. Participants were initially allowed to skip any item, however, following an interim analysis on 27th April, a high proportion of missing data for country and CA type was noted. As these data were crucial to the research question, these two items were subsequently made mandatory.

Patient and Public Involvement

People with experience in caring for or teaching children with CAs contributed to the development of the survey. These were (a) three parents of children with CAs who also run patient organisations/charities relevant to their child's condition (Down syndrome, spina bifida, metachromatic leukodystrophy), (b) a clinical geneticist who works closely with a number of parent organisations, and (c) a teacher of children with special educational needs and disabilities. Each

For peer review only - http://bmjopen.bmj.com/site/about/guidelines.xhtml

individual commented on an early draft of the survey, including the overall content of the survey, and the wording of questions and response options. This feedback was reviewed by the research team and relevant modifications were made to the draft survey to address it.

Findings from the study will be shared with members of the public, parents and carers, healthcare professionals and relevant stakeholders via scientific publications, lay reports, social media and conferences.

Data analysis

Descriptive statistics were conducted using Stata 17.0 software.[34] Data were checked to ensure that answers were consistent (e.g. identifying if a mother replying that she is 20-25 years old is retired). Outcomes scored on 4-point Likert scales (very much/satisfied vs. other responses) were dichotomised and outcomes scored on a 5-point Likert scale were collapsed into three categories by merging the two lowest response options (much worse, somewhat worse) and the two highest response options (much better, somewhat better). Data were modelled using multivariate logistic regressions and ordinal logistic regressions which included the child's anomaly type, and parent's country of residence, age and education level. The impact of country and anomaly type on outcomes was explored, choosing the largest categories as the comparator groups (Poland and CHD). For age and education, categorical data were collected. For the analysis each variable was re-coded into three groups: age (<30 years; 31-40 years, >40 years), education (formal education/technical training until 16 or 18 years; university degree; post-graduate degree). Age and education were included in our regression models as ordinal variables. To control for multiple comparisons, the alpha level was adjusted to $p < 0.01$ for all analyses. It was unlikely that data were missing at random so more sophisticated multiple imputation techniques were not adopted.

We aimed to recruit 80 participants per country which would have resulted in a power of 80% to determine that a country with 20% of participants replying category 4 (very much/satisfied) was statistically significantly different at the 95% level of significance from a country with 40% of participants replying category 4. Owing to delays in obtaining ethics approvals, this target was not met within the timescales for some countries. Data were presented by country if these were available for at least 50 participants. Where there were <50 participants, data were combined into an 'other European country' group (termed henceforth as *Other EU*), which included participants from a heterogenous group of countries. Due to similarities in survey responses, geographical location, and language, data for Belgium (n=46) and the Netherlands (n=28) were combined into a single group. For CAs, data were categorised according to the four anomalies, and a separate category created for children with Down syndrome and a CHD, a common co-morbidity.[35] There

For peer review only - http://bmjopen.bmj.com/site/about/guidelines.xhtml

were too few participants to create meaningful categories for children who had other combinations of the four anomalies (n=15), and these were excluded from the analysis.

Given that a multi-modal online recruitment strategy was used, it was not possible to estimate how many potential participants the survey reached in order to calculate response rates.[36] Submission rates (number of participants who started the survey/number who completed and submitted the survey)[37], and of those who submitted their survey, we calculated item-level response rates (proportion of participants completing each item)[38] are reported.

RESULTS

Participant characteristics

1,298 parents across Europe accessed the survey, of whom 1,109 (85%) submitted their responses. The submission rate ranged from 78% in Italy to 92% in Germany and Belgium. A further 123 (9.5%) submitted forms were not included in the analysis as country data were missing (n=80), CA data were missing (n=24), participants were from non-European countries (n=4) or participants specified different combinations of the four anomaly types (n=15). Item-level response rates were above 98% across all outcome variables.

Participants lived in Poland (n=476), the UK (n=120), Germany (n=97), Belgium/Netherlands (n=74), Croatia (n=68), Italy (n=59). The Other EU group (n=92) comprised participants from: Denmark (n=39), Portugal (n=23), Spain (n=16), Ireland (n=5), Bulgaria (n=2), Albania (n=1), Cyprus (n=1), Lithuania (n=1), Norway (n=1), Romania (n=1), Sweden (n=1), Ukraine (n=1). Most respondents were mothers (92%), aged 31-40 years (71%), and in full- or part-time employment (59%) (Table 1). In terms of education, 40% of participants had received formal education up to 16 or 18 years or technical training, 49% had a university degree, and 11% a post-graduate degree. Few participants had lived in their country of residence for <10 years (6%).

Child characteristics

The largest CA group was CHD (n=327; 33%). Other children were diagnosed with Down syndrome (n=262; 26%), a cleft lip (n=230; 23%), spina bifida (n=112; 11%) and Down syndrome with a CHD (n=55; 6%). In terms of co-morbidities, 25% of children had another CA, and 43% had another health condition. The most common age category was 1-3 years (35%) and there was a slightly higher proportion of male children (56%). Just over a third of children attended school (36%), whereas 62% were not yet of school age, and 2% were either home-schooled or unable to be schooled due to their health status.

For peer review only - http://bmjopen.bmj.com/site/about/guidelines.xhtml

(a) Provision of healthcare across countries

Cancelled or postponed appointments

Cancellations or postponements of routine appointments were reported by 68% (623/920) of the whole sample, by 53% (427/803) for planned tests or procedures, and by 26% (121/609) for planned surgeries. The UK and Poland had the largest proportions of parents reporting cancelled or postponed appointments for each category (Figure 1). For routine appointments and planned tests/procedures, proportions were significantly lower in Germany, Croatia, Belgium/Netherlands, and the Other EU group compared with Poland (Table 2). For planned surgeries, all countries except the UK had a significantly lower proportion of cancelled or postponed appointments than Poland (full regression findings are available in the supplementary file).

Table 2 Proportion of participants reporting 'cancelled or postponed' routine appointments, planned tests or procedures, and planned surgeries, by country.

Country	Routine appointments (N†=920)		Planned tests or procedures (N†=803)		Planned surgeries (N†=609)	
	Unadjusted % [95% CI]	*Adjusted** % [95% CI]	*Unadjusted* % [95% CI]	*Adjusted** % [95% CI]	*Unadjusted* % [95% CI]	*Adjusted** % [95% CI]
Poland	79 [75-83]	79 [75-83]	66 [61-70]	65 [60-70]	37 [31-43]	35 [29-41]
UK	88 [82-94]	86 [80-93]	67 [58-76]	67 [57-76]	31 [20-41]	33 [22-44]
Germany	29 [19-38]	31 [21-42]	16 [8-24]	18 [9-27]	7 [1-14]	8 [1-15]
Croatia	46 [34-58]	44 [32-56]	36 [23-49]	36 [23-49]	14 [3-24]	13 [3-24]
Italy	69 [57-81]	70 [58-82]	52 [38-66]	54 [40-68]	9 [1-18]	11 [0-20]
Belgium/Netherlands	34 [23-46]	39 [27-51]	20 [9-31]	23 [11-34]	17 [7-27]	16 [6-25]
Other EU	56 [46-67]	57 [47-67]	43 [32-53]	43 [32-53]	12 [4-19]	12 [4-20]

†Total number of participants excluding 'not applicable' responses. Missing data: routine appointments (9 participants), planned tests or procedures (8 participants), planned surgeries (5 participants).
*Adjusted by congenital anomaly type, parental age, and education level.
CI = confidence intervals

Virtual appointments (by telephone or online)

Overall, 61% (544/891) of participants reported that their child's face-to-face appointments had been rescheduled as virtual appointments. This proportion was highest in the UK (87%), significantly higher than in Poland (71%). In all other countries this proportion was significantly lower than Poland (Table 3).

For peer review only - http://bmjopen.bmj.com/site/about/guidelines.xhtml

Table 3 Proportion* of participants reporting appointments rescheduled as virtual, virtual appointments rated as 'poor', and problems accessing medication, by country.

Country	Appointments rescheduled as virtual (N†=891)		Virtual appointments rated as 'poor' (N†=552)		Problems accessing medication (N†=713)	
	Unadjusted % [95% CI]	*Adjusted** % [95% CI]	*Unadjusted* % [95% CI]	*Adjusted** % [95% CI]	*Unadjusted* % [95% CI]	*Adjusted** % [95% CI]
Poland	72 [68-76]	71 [67-75]	37 [32-42]	37 [32-43]	34 [29-39]	34 [29-39]
UK	87 [81-93]	87 [81-93]	21 [13-29]	21 [13-29]	43 [33-54]	42 [32-52]
Germany	24 [15-33]	25 [16-35]	0 [0-17]	0 [0-17]	6 [1-12]	7 [1-14]
Croatia	46 [33-59]	46 [33-58]	15 [2-29]	17 [2-31]	5 [0-11]	4 [0-10]
Italy	34 [21-47]	37 [23-50]	29 [8-51]	27 [7-48]	14 [3-25]	14 [3-25]
Belgium/Netherlands	28 [17-40]	34 [22-46]	6 [0-18]	5 [0-15]	19 [8-29]	19 [8-29]
Other EU	50 [40-60]	52 [42-62]	23 [10-35]	22 [10-34]	9 [2-16]	8 [2-14]

†Total number of participants excluding 'not applicable' responses. Missing data: appointments rescheduled as virtual (8 participants), virtual appointments rated as 'poor' (11 participants), problems accessing medication (8 participants).

*Adjusted by congenital anomaly type, parental age and education level.

CI = confidence intervals

Overall, 29% (159/541) of participants reported their child's virtual appointments as being of 'poor' quality overall. This proportion was highest in Poland (37%), and significantly lower in the UK (21%), Belgium/Netherlands (5%), and Germany (0%) (Table 3). There was a significant impact of education level on ratings, whereby more highly educated participants were less likely to rate the overall quality of their virtual appointments as 'poor' (Odds Ratio [OR] = 0.55, 95% CI: 0.41-0.77; p=0.000).

Access to medication

Overall, 26% (182/705) of participants reported some problems accessing medication for their child during the pandemic. This proportion was highest in the UK (42%) and Poland (34%) (Table 3). Italy (14%), the Other EU group (8%), Germany (7%), and Croatia (4%) all had significantly fewer participants reporting problems compared with Poland (Table 3).

(b) Impact on the child's health and wellbeing across countries

Overall, 30% (221/749) of participants reported that changes to their child's treatment during the pandemic had moderately to severely compromised their child's health. This figure was significantly higher in Poland (43%) compared with the UK (28%), the Other EU group (20%), Croatia (15%), Italy (12%), Germany (12%), and Belgium/Netherlands (7%) (Figure 2).

The majority of participants rated their child's physical health (68%; 634/927) and emotional wellbeing (56%; 515/927) as being 'about the same' as it was prior to the pandemic. Overall, there was a greater proportion of participants who rated their child's emotional wellbeing as 'worse' (35%; 319/927) compared to 'worse' ratings for physical health (17%; 162/927).

For peer review only - http://bmjopen.bmj.com/site/about/guidelines.xhtml

There was a significant impact of country on ratings for physical health, with all countries less likely to rate their child's physical health as 'worse' than before the pandemic compared with Poland (Figure 3). Ratings for the impact of COVID-19 on emotional wellbeing were similar across countries.

(c) Support for parents across countries

Overall, 23% (220/957) of participants reported that they would have liked more support during the pandemic 'very much'. This proportion was highest in Poland (30%), and significantly lower in Croatia (14%), Belgium/Netherlands (9%), and the Other EU group (11%) (Figure 4). In terms of the source of support, satisfaction ratings were lowest for support from medical sources, and highest for people that participants had close relationships with, such as their partner (Table 4).

For peer review only

For peer review only - http://bmjopen.bmj.com/site/about/guidelines.xhtml

Table 4 Proportion* of participants reporting that they were 'very satisfied' with the support they received from each source, by country.

Country	GP (N†=775)	Specialist doctor/nurse (N†=792)	Partner (N†=868)	Friends/family (N†=883)	Parents of children with same condition (N†=637)	Patient organisations (N†=510)	Schools (N†=369)
	% [95% CI]	% [95% CI]	% [95% CI]	% [95% CI]	% [95% CI]	% [95% CI]	% [95% CI]
Poland	26 [21-30]	34 [27-37]	69 [65-74]	61 [56-66]	66 [61-71]	56 [50-62]	27 [20-34]
UK	25 [16-34]	39 [29-49]	79 [71-86]	49 [40-58]	51 [41-61	38 [27-48]	47 [34-60]
Germany	85 [76-94]	84 [76-93]	89 [81-97]	73 [62-85]	60 [42-79]	59 [38-80]	39 [17-61]
Croatia	41 [28-53]	46 [34-59]	80 [1-90]	64 [52-75]	64 [51-76]	36 [21-51]	19 [2-36]
Italy	44 [29-59]	31 [16-45]	71 [58-84]	47 [32-61]	44 [26-61]	33 [18-49]	42 [27-57]
Belgium/ Netherlands	57 [44-72]	59 [46-71]	66 [54-77]	49 [37-61]	33 [17-50]	14 [0-27]	36 [20-52]
Other EU	45 [33-57]	45 [34-56]	70 [60-79]	52 [42-63]	56 [43-68]	38 [26-51]	33 [19-46]
Total	37 [33-40]	42 [39-45]	72 [70-75]	58 [55-61]	60 [56-64]	46 [42-50]	34 [29-38]

*Adjusted by congenital anomaly type, parental age, and education level. Unadjusted proportions are not included in this table.
†Total number of participants completing the item, excluding 'not applicable' responses. Missing data: GP (n=10), specialist doctor/nurse (n=16), partner (n=11), friends/family (n=14), parents of children with same condition (n=17), patient organisations (n=18), schools (n=35).
CI = confidence interval; GP = general practitioner

For peer review only

Medical sources

The UK and Poland had the lowest proportion of 'very satisfied' ratings for GPs, 25% and 26% respectively (Table 4). Compared to Poland, ratings were significantly higher in Germany (85%) and the Other EU group (45%). Italy and Poland had the lowest 'very satisfied' ratings for specialist doctors/nurses, 31% and 32%, respectively. Compared to Poland, ratings were significantly higher in Germany (84%) and the Other EU group (45%) (Table 4).

Organisations

The highest proportion of 'very satisfied' ratings for patient organisations were for parents in Germany (59%) and Poland (56%) (Table 4). Compared with Poland, these satisfaction ratings were significantly lower in the UK (38%) and Belgium/Netherlands (14%). The UK had the highest proportion of participants who were 'very satisfied' with support from their child's school (47%), however, there were no significant country related effects.

Close relationships

Poland had the highest proportion of 'very satisfied' ratings for support from parents of other children with the same health condition (66%), significantly higher than the UK (51%) and Belgium/Netherlands (33%) (Table 4). There were no significant differences in satisfaction ratings for support from 'partner' or 'friends/family' across countries.

(d) Outcomes across CA types

There were few differences across CA types, with significant differences only found for items relating to the provision of healthcare. In summary, parents of children with CHD (43%) reported a significantly lower proportion of 'cancelled or postponed' tests/procedures compared with parents of children with spina bifida (65%) and Down syndrome (alone) (62%) (Table 5). The CHD group also reported significantly fewer rescheduled appointments (49%) compared with the Down syndrome with CHD (80%), Down syndrome (72%), and spina bifida (70%) groups (Table 5). A lower proportion of parents of children with a cleft lip (17%) reported problems accessing medication compared with the CHD group (34%) (Table 5).

For peer review only - http://bmjopen.bmj.com/site/about/guidelines.xhtml

Table 5 Proportion of participants reporting 'cancelled or postponed' planned tests/procedures, appointments rescheduled as virtual, virtual appointments rated as 'poor', and problems accessing medication, by CA type.

**Congenital anomaly**	**Planned tests or procedures (N†=803)**		**Appointments rescheduled as virtual (N†=891)**		**Virtual appointments rated as 'poor' (N†=552)**		**Problems accessing medication (N†=713)**	
	Unadjusted	*Adjusted**	*Unadjusted*	*Adjusted**	*Unadjusted*	*Adjusted**	*Unadjusted*	*Adjusted**
	% [95% CI]	% [95% CI]	% [95% CI]	% [95% CI	% [95% CI]	% [95% CI]	% [95% CI]	% [95% CI]
CHD	42 [36-47]	43 [37-49]	46 [41-52]	49 [44-55]	36 [29-44]	36 [28-44]	31 [25-36]	34 [21-40]
Cleft lip	48 [41-55]	51 [44-58]	52 [45-58]	56 [49-62]	33 [24-42]	29 [21-38]	16 [9-22]	17 [10-23]
Spina bifida	64 [54-73]	65 [56-73]	66 [58-75]	70 [62-77]	30 [19-40]	27 [17-37]	30 [21-39]	28 [19-36]
Down syndrome	67 [61-73]	62 [55-68]	76 [71-81]	72 [67-78]	28 [21-34]	29 [22-36]	27 [20-33]	24 [18-30]
Down syndrome with CHD	60 [47-74]	55 [40-68]	83 [73-93]	80 [69-91]	33 [18-47]	28 [13-42]	20 [8-31]	17 [6-27]

†Total number of participants excluding 'not applicable' responses. Missing data: planned tests/procedures (n=8), appointments rescheduled as virtual (n=8), virtual appointments rated as 'poor' (n=11), problems accessing medication (n=8).

*Adjusted by parental country of residence, age, and education level.

CA = congenital anomaly; CI = confidence intervals; CHD=congenital heart defects

For peer review only - http://bmjopen.bmj.com/site/about/guidelines.xhtml

DISCUSSION

Main findings

This study provides a snapshot of the healthcare experiences of children with CAs, and their caregivers' experiences of support across Europe, one year into the COVID-19 pandemic. Overall, many participants reported disruptions to their child's routine care, which appeared to have an impact on the health of some children. Compared with non-medical organisations and parents' close relationships, parents were least satisfied with support from GPs and specialist doctors/nurses, which was particularly poor in Poland and the UK. There were also regional differences in the proportions of parents reporting disruptions to healthcare, which again appeared most severe in Poland and the UK. Few differences were found in outcomes according to CA type, suggesting that the geographical location of participants had more of an influence on healthcare experiences than the child's specific health condition.

Whilst acknowledging that a range of factors may underpin differences across countries (such as reductions in hospital visits to minimise infections), a possible hypothesis is that these are indicative of existing vulnerabilities within local healthcare systems, with lower resourced systems being less able to meet the needs of patients during the pandemic. In relation to the healthcare workforce, figures from the Organisation for Economic Co-operation and Development (OECD) in 2018 indicate that Poland had the lowest numbers of practising doctors per head in Europe (2.3/1,000), closely followed by the UK (2.8/1,000).[39] In contrast, Germany had one of the highest numbers per head in Europe (4.4/1,000).[39] Among European countries, Poland, Italy and the UK also had below average numbers of practicing nurses per head, a factor associated with patient satisfaction with care,[40] which ranged from 5.1-7.8/1,000.[39] In comparison, the Netherlands, Belgium, and Germany all had above average figures, 11.1, 11.2 and 13.1/1,000, respectively. A larger number of healthcare workers is likely to have helped with increased demand during the pandemic, and helped mitigate the consequences of staff sickness.[39] Other factors that may account for regional differences include our method of recruitment and the severity of local restrictions. Participants were recruited with a consistent strategy across countries, however, some had a greater number of organsations who advertised the study and some were able to request that these organisations advertise the survey more frequently (for example in Poland). Recruitment periods also differed, ranging from 18 weeks in the UK and Poland to only 6.5 weeks in Italy (Table 1). As people are more likely to respond to surveys which are highly salient to their experiences,[41] one might expect parents experiencing more challenges during the pandemic to have been more likely to respond. However, our survey was a general survey about parents' information and support needs, with only one subsection relating to

For peer review only - http://bmjopen.bmj.com/site/about/guidelines.xhtml

the pandemic, so this is unlikely. It is difficult to estimate the extent to which variations in the depth and duration of COVID-19 containment strategies might have influenced cross-country variations in outcomes. Containment strategies varied both regionally, within each country, and internationally, during the survey recall period (January 2020-July 2021). In addition, all countries experienced a period of full lockdown during this time frame, making these other variations less pertinent.

The use of virtual healthcare appointments during the pandemic meant parental concerns about cancellations could be addressed, whilst limiting their exposure to SARS-CoV-2.[19] Around 60% of our sample reported that they had a face-to-face appointment re-scheduled as virtual. This was higher than other in other studies conducted with pediatric patients, which found that 11%[18] and 20%[42] of participants reporting re-scheduled appointments. However, these studies were conducted during the first wave of the pandemic (April-May 2020), a year before our survey was delivered, so this rise is not unexpected. The quality of virtual appointments was rated as 'fair-excellent' by two-thirds of our sample, whereas one third rated them as 'poor'. This is in line with findings from a similar survey which found that 68% of CHD parents and patients described their virtual appointments as "adequate".[42] In contrast, other studies conducted within specialist pediatric centres found higher satisfaction ratings,[19,43] such as 87% mean satisfaction ratings for virtual pediatric appointments.[44] This increase, however, may be due to a slight social-desirability bias as parents were asked to rate satisfaction by staff within the specialist centres, whereas in our survey the recruiters were not involved in the child's care. Of note is our finding whereby participants with a lower level of education were more likely to rate virtual appointments as poor.

Strengths and limitations

This study surveyed parents and carers of children with different CA types within several countries, including a broad range of experiences. We recruited a large sample of parents and carers overall, and the proportion of each CA type reflects the relative number of live births with each CA in Europe.[45] Although the survey was shared widely, the use of convenience sampling means there is a risk of selection bias, and the views and experiences of this sample may not be representative of all parents and carers of children with CAs. The use of social media to recruit participants may have excluded people living with 'digital poverty' and people who don't tend to engage with these types of organisations, whose experiences may differ from this sample. When considering recruitment figures within each country these were mostly small. The survey was developed with input from parents of children with a CA, however, we were unable to conduct a full pilot of the final version. Great care was taken to avoid leading questions, ambiguity or complex language, however, it is possible that there may have been some issues with the wording or content of survey items.

For peer review only - http://bmjopen.bmj.com/site/about/guidelines.xhtml

Implications and future research

Our survey findings are important and provide useful insights into the provision of care for children with CAs across Europe during the first year of the pandemic. Findings highlight potential weaknesses of healthcare systems in some countries and suggest that long-term systemic action is required to improve patient experiences and outcomes. The situation appears particularly problematic in the UK and Poland, which may benefit from increased resources to provide for this vulnerable group of patients. Patient organisations and charities provide an invaluable source of knowledge and support to parents of children with CAs, and these should be supported, especially in countries where medical capacity to meet patients' needs may be stretched.

As with many other patient groups, it is clear that the COVID-19 pandemic has had an impact on the experiences of children living with a serious health condition and their families.[20,46,47] This particular survey suggests disruptions to care for children, with potential impacts on the child's health and well-being. Considering the limitations of this study, it will be important to further investigate the impact of the COVID-19 pandemic on the delivery of paediatric services across Europe using population-based data. With the proliferation of telemedicine to deliver care during the pandemic,[44] exploring the reasons why these virtual strategies were lacking for some parents (particularly those with a lower level of education) is important to ensure optimal parental satisfaction with future care and support from medical professionals.

CONCLUSION

The COVID-19 pandemic continues to put pressure on healthcare systems worldwide. Our survey findings highlight disruptions to the delivery of care across Europe, particularly in the UK and Poland, which raises questions about the ability of the healthcare systems within these countries to meet the needs of children with CAs and their families, and a need for increased resources.

ACKNOWLEDGEMENTS

The authors are hugely grateful to all the parents and carers who took part in the study. We thank the following people for their support in developing the survey and its dissemination in Poland: Dominika Madaj-Solberg (Spina Foundation, Katowice, Poland), Tomek i Kasia Grybek (Borys the Hero Foundation, Gdańsk, Poland), Halina Grzymisławska-Słowińska (Fundacja TAK dla Samodzielności, Poznań, Poland), Anna Latos (Bydgoszcz, Poland), Prof. Jolanta Wierzba (Med.Univ. Gdańsk, Poland), Prof. Robert Śmigiel (Med. Univ. Wrocław, Poland), Prof. Olga Haus (Coll.Med. UMK, Bydgoszcz, Poland), and Dorota Trześniewska (Poznań, Poland). We thank the following people

For peer review only - http://bmjopen.bmj.com/site/about/guidelines.xhtml

and organisations for advertising the survey across Europe: The Cleft Lip and Palate Association, The Children's Heart Federation, International Federation for Spina Bifida and Hydrocephalus, Children's Heartbeat Trust, Down's Syndrome Association, and Down Syndrome International, Dr. Nadia Assanta (Fondazione Toscana Gabriele Monasterio), Dr. Giada Cavazzuti (Associazione “Un cuore, un mondo”), Dr. Elisabetta Lapi, Dr. Antonella Falugiani (Associazione “Trisomia 21 Onlus”), Dr. Alessandro Giacomina, Dr. Marina Rossi (AOU Pisana), Jürgen Wolters (Arbeitsgemeinschaft Spina Bifida und Hydrocephalus, ASBH), Dr Annett Lambrecht (Department of Pediatric Cardiology, University Hospital Magdeburg), Dr Christian Zahl (Department of Oral and Maxillofacial Surgery, University Hospital Magdeburg), Hjerteforeningens børneklub (Kid’s Heart Association Club), Rygmarvsbrokforeningen (Spina Bifida and Hydrocephalus Association), Downs syndrom Danmark (Danish Down Syndrome), Landsforeningen Læbe- Ganespalte (Cleft Lip and Palate Association), Pais21 (Down Portugal/ Down Syndrom Parents Association Pais 21), Associação Spina Bifida e Hidrocefalia de Portugal (Spina Bifida and Hidrocephalus Portugal Association), Associação Coração Feliz (Happy Heart Association), Associação Portuguesa dos Amigos das Crianças Portadoras de Fendas Lábio-Palatinas (Portugese Association of Children with Lip-Palatine Clefts), The Foundation for the Promotion of Health and Biomedical Research of Valencia Region (FISABIO), Universitair Ziekenhuis Antwerpen (University Hospital of Antwerp), Vereniging voor Aangeboren Gelaatsafwijkingen (Association for Congenital Facial Defects), Dr Annick Laridon - Het Centrum voor Ontwikkelingsstoornissen (Centre for Developmental Disorders), Mario Sel - Spina Bifida Hydrocephalus Belgium, Hrvatski savez za rijetke bolesti (Rare Diseases Croatia), Veliko srce malom srcu (A Big Heart For Little Heart), Hrvatska zajednica za Down sindrom (Croatian Down Syndrome Association), Udruga roditelja djece s rascjepom usne i/ili nepca OSMIJEH (Association of Children With Cleft Lip With/Without Cleft Palate), Udruga Aurora- Udruga roditelja i djece sa spinom bifidom (Aurora Association- Association of Parents and Children with Spina Bifida), Patientenvereniging Aangeboren Hartaandoeningen (Congenital Heart Disease Association), De 'Stichting Downsyndroom' (Down Syndrome Association). We are very grateful to Esben Garne Holm and Juan Rico for supporting the translation of the survey.

ETHICS APPROVAL

Ethics approval for the study was granted by the St George’s (University of London) Research Ethics Committee on 18th December 2020 (reference number: 2020.0311). In Poland, ethics approval was granted on 10th December 2020 by the Bioethics Committee at the Poznań University of Medical Sciences (reference number: 882/20). In Croatia, ethics approval was granted on 10th December 2020 by the Ethics Committee of the Children’s Hospital Zagreb (Protocol No: 02-23/43-1-20 Zagreb).

For peer review only - http://bmjopen.bmj.com/site/about/guidelines.xhtml

In Spain, ethics approval was granted on 21st December 2020 by the Clinical Investigation Ethics Committee of the "Dirección General de Salud Pública y Centro Superior de Investigación en Salud Pública" (reference number: 20201221/05). In Belgium, ethics approval was granted on 1st March 2021 by the Ethics Committee of the University Hospital of Antwerp (reference: 21/06/084). In Portugal, ethics approval was granted on 16th March by the Ethics Committee of the National Institute of Health Doutor Ricardo Jorge (CES-INSA). In Germany, ethics approval was granted on 15th April 2021 by the Medical Faculty of the Otto-von-Guericke-University Magdeburg Research Ethics Committee (reference number: 44/21). In Italy, ethics approval was granted on 14th June 2021 by the Research Ethics and Integrity Committee of the National Research Council Institute of Clinical Physiology in Pisa (CNR-INF) (protocol number 0065527/2019). No local ethics approvals were required in Denmark (Lillebaelt Hospital – University Hospital of Southern Denmark) or the Netherlands (University Medical Center Groningen).

DATA AVAILABILITY STATEMENT

The datasets analysed during the current study are available from the corresponding author on reasonable request.

CONTRIBUTERS

ALB conceptualised the study. ALB, EM, and JKM contributed to the study design. EM led the survey development, translation, and recruitment of participants. EM and JKM conducted the data analysis. ALB, JKM, and JR critically revised the manuscript. AJD, IB, CCC, EDH, EG, EM, LG, AJS, RT, CMD, LRL, CNP, AJN, AN, LO, LPR, AP, and AR oversaw the translation of the survey and recruited participants. EM drafted the manuscript. All authors contributed to, read and approved the final manuscript.

FUNDING

This project has received funding from the European Union's Horizon 2020 research and innovation programme under grant agreement No 733001. Start date: 1 Jan 2017. Duration: 5 years and 5 months. The views presented here are those of the authors only, and the European Commission is not responsible for any use that may be made of the information presented here.

COMPETING INTERESTS

All authors have completed the ICMJE uniform disclosure form at http://www.icmje.org/disclosure-of-interest/ and declare: all authors had financial support from the European Union's Horizon 2020 research and innovation programme for the submitted work; no financial relationships with any

For peer review only

organisations that might have an interest in the submitted work in the previous three years; no other relationships or activities that could appear to have influenced the submitted work.

TRANSPARENCY DECLARATION

The corresponding author affirms that the manuscript is an honest, accurate, and transparent account of the study being reported; that no important aspects of the study have been omitted; and that any discrepancies from the study as planned (and, if relevant, registered) have been explained.

DISSEMINATION TO STUDY PARTICIPANTS AND RELATED PATIENT AND PATIENT COMMUNITIES

Findings from the study will be shared with members of the public, parents and carers, healthcare professionals and relevant stakeholders via scientific publications, lay reports, social media, and conferences.

For peer review only

REFERENCES

1 Smolic, S., Cipin, I. & Medimurec, P. Access to healthcare for people aged 50+ in Europe during the COVID-19 outbreak. *European Journal of Ageing*, 1-17, doi:10.1007/s10433-021-00631-9 (2021).

2 Cena, L., Rota, M., Calza, S. *et al.* Estimating the Impact of the COVID-19 Pandemic on Maternal and Perinatal Health Care Services in Italy: Results of a Self-Administered Survey. *Frontiers in Public Health* **9**, 701638, doi:10.3389/fpubh.2021.701638 (2021).

3 van Veenendaal, N. R., Deierl, A., Bacchini, F. *et al.* Supporting parents as essential care partners in neonatal units during the SARS-CoV-2 pandemic. *Acta Paediatr* **110**, 2008-2022, doi:10.1111/apa.15857 (2021).

4 Gardner, T. & Fraser, C. Elective care: how has COVID-19 affected the waiting list? London: The Health Foundation. Available from: https://bit.ly/3EKqvRL [accessed 1 Nov 2021] (2021).

5 Chiumento, A., Baines, P., Redhead, C. *et al.* Which ethical values underpin England's National Health Service reset of paediatric and maternity services following COVID-19: a rapid review. *BMJ Open* **11**, e049214, doi:10.1136/bmjopen-2021-049214 (2021).

6 Colvin, L. & Bower, C. A retrospective population-based study of childhood hospital admissions with record linkage to a birth defects registry. *BMC Pediatr* **9**, 32, doi:10.1186/1471-2431-9-32 (2009).

7 Rosano, A., Botto, L. D., Botting, B. *et al.* Infant mortality and congenital anomalies from 1950 to 1994: an international perspective. *Journal of Epidemiology and Community Health* **54**, 660-666, doi:10.1136/jech.54.9.660 (2000).

8 Department of Health. *National framework for children and young peoples continuing care*. (Department of Health, London, 2016).

9 Razzaghi, H., Dawson, A., Grosse, S. D. *et al.* Factors associated with high hospital resource use in a population-based study of children with orofacial clefts. *Birth Defects Res A Clin Mol Teratol* **103**, 127-143, doi:10.1002/bdra.23356 (2015).

10 Fitzgerald, P., Leonard, H., Pikora, T. J. *et al.* Hospital admissions in children with down syndrome: experience of a population-based cohort followed from birth. *PLoS ONE* **8**, e70401, doi:10.1371/journal.pone.0070401 (2013).

11 Bishop, C. F., Small, N., Parslow, R. *et al.* Healthcare use for children with complex needs: using routine health data linked to a multiethnic, ongoing birth cohort. *BMJ Open* **8**, e018419, doi:10.1136/bmjopen-2017-018419 (2018).

12 Alanazi, A. F., Naser, A. Y., Pakan, P. *et al.* Trends of Hospital Admissions Due to Congenital Anomalies in England and Wales between 1999 and 2019: An Ecological Study. *International Journal of Environmental Research and Public Health* **18**, 11808, doi:10.3390/ijerph182211808 (2021).

13 Ludvigsson, J. F. Systematic review of COVID-19 in children shows milder cases and a better prognosis than adults. *Acta Paediatr* **109**, 1088-1095, doi:10.1111/apa.15270 (2020).

14 Malle, L., Gao, C., Hur, C. *et al.* Individuals with Down syndrome hospitalized with COVID-19 have more severe disease. *Genet Med* **23**, 576-580, doi:10.1038/s41436-020-01004-w (2021).

15 Clift, A. K., Coupland, C. A. C., Keogh, R. H. *et al.* COVID-19 Mortality Risk in Down Syndrome: Results From a Cohort Study of 8 Million Adults. *Ann Intern Med* **174**, 572-576, doi:10.7326/M20-4986 (2021).

16 Malviya, A. & Yadav, R. COVID -19 pandemic and paediatric population with special reference to congenital heart disease. *Indian Heart J* **72**, 141-144, doi:10.1016/j.ihj.2020.06.001 (2020).

17 Madhusoodhan, P. P., Pierro, J., Musante, J. *et al.* Characterization of COVID-19 disease in pediatric oncology patients: The New York-New Jersey regional experience. *Pediatr Blood Cancer* **68**, e28843, doi:10.1002/pbc.28843 (2021).

For peer review only - http://bmjopen.bmj.com/site/about/guidelines.xhtml

18 Poppe, M., Aguiar, B., Sousa, R. *et al.* The Impact of the COVID-19 Pandemic on Children's Health in Portugal: The Parental Perspective. *Acta Med Port* **34**, 355-361, doi:https://dx.doi.org/10.20344/amp.14805 (2021).

19 Darr, A., Senior, A., Argyriou, K. *et al.* The impact of the coronavirus (COVID-19) pandemic on elective paediatric otolaryngology outpatient services - An analysis of virtual outpatient clinics in a tertiary referral centre using the modified paediatric otolaryngology telemedicine satisfaction survey (POTSS). *Int J Pediatr Otorhinolaryngol* **138**, 110383, doi:https://dx.doi.org/10.1016/j.ijporl.2020.110383 (2020).

20 Wray, J., Pagel, C., Chester, A. H. *et al.* What was the impact of the first wave of COVID-19 on the delivery of care to children and adults with congenital heart disease? A qualitative study using online forums. *BMJ Open* **11**, e049006, doi:10.1136/bmjopen-2021-049006 (2021).

21 The Cleft Lip and Palate Association. Summer Survey 2020: The Results. Available from: https://www.clapa.com/news-item/summer-survey-2020-the-results/ [accessed 2 Nov 2021]. (2020).

22 Marino, L. V., Wagland, R., Culliford, D. J. *et al.* "No Official Help Is Available"-Experience of Parents and Children With Congenital Heart Disease During COVID-19. *World Journal for Pediatric & Congenital Heart Surgery* **12**, 500-507, doi:https://dx.doi.org/10.1177/21501351211007102 (2021).

23 Cousino, M. K., Pasquali, S. K., Romano, J. C. *et al.* Impact of the COVID-19 pandemic on CHD care and emotional wellbeing. *Cardiol Young* **31**, 822-828, doi:10.1017/S1047951120004758 (2021).

24 Stewart, A., Smith, C. H., Eaton, S. *et al.* COVID-19 pandemic experiences of parents caring for children with oesophageal atresia/tracheo-oesophageal fistula. *BMJ Paediatr Open* **5**, e001077, doi:10.1136/bmjpo-2021-001077 (2021).

25 Mongru, R., Rose, D., Costelloe, C. *et al.* Retrospective analysis of North West London healthcare utilisation by children during the COVID-19 pandemic. *BMJ Paediatrics Open* **6**, e001363, doi:10.1136/bmjpo-2021-001363 (2022).

26 Kruizinga, M. D., Peeters, D., van Veen, M. *et al.* The impact of lockdown on pediatric ED visits and hospital admissions during the COVID19 pandemic: a multicenter analysis and review of the literature. *Eur J Pediatr* **180**, 2271-2279, doi:10.1007/s00431-021-04015-0 (2021).

27 Nicholson, E., McDonnell, T., Conlon, C. *et al.* Parental Hesitancy and Concerns around Accessing Paediatric Unscheduled Healthcare during COVID-19: A Cross-Sectional Survey. *Int J Environ Res Public Health* **17**, 11, doi:https://dx.doi.org/10.3390/ijerph17249264 (2020).

28 Liguoro, I., Pilotto, C., Vergine, M. *et al.* The impact of COVID-19 on a tertiary care pediatric emergency department. *Eur J Pediatr* **180**, 1497-1504, doi:https://dx.doi.org/10.1007/s00431-020-03909-9 (2021).

29 Dorfman, L., Nassar, R., Binjamin Ohana, D. *et al.* Pediatric inflammatory bowel disease and the effect of COVID-19 pandemic on treatment adherence and patients' behavior. *Pediatric Research* **90**, 637-641, doi:https://dx.doi.org/10.1038/s41390-020-01312-6 (2021).

30 Morris, J. K., Garne, E., Loane, M. *et al.* EUROlinkCAT protocol for a European population-based data linkage study investigating the survival, morbidity and education of children with congenital anomalies. *BMJ Open* **11**, e047859, doi:10.1136/bmjopen-2020-047859 (2021).

31 Cuschieri, S. The STROBE guidelines. *Saudi J Anaesth* **13**, S31-S34, doi:10.4103/sja.SJA_543_18 (2019).

32 Kuliś, D., Bottomley, A., Velikova, G. *et al.* EORTC Quality of Life Group Translation Procedure (4th edition). Available from: https://www.eortc.org/app/uploads/sites/2/2018/02/translation_manual_2017.pdf [accessed 14 Nov 2021]. (2017).

For peer review only

33 Harris, P. A., Taylor, R., Thielke, R. *et al.* Research electronic data capture (REDCap)--a metadata-driven methodology and workflow process for providing translational research informatics support. *J Biomed Inform* **42**, 377-381, doi:10.1016/j.jbi.2008.08.010 (2009).

34 Stata Statistical Software: Release 17 (College Station, TX: StataCorp LLC, 2021).

35 Leirgul, E., Fomina, T., Brodwall, K. *et al.* Birth prevalence of congenital heart defects in Norway 1994–2009 - a nationwide study. *Am Heart J* **168**, 956-964 (2014).

36 McRobert, C. J., Hill, J. C., Smale, T. *et al.* A multi-modal recruitment strategy using social media and internet-mediated methods to recruit a multidisciplinary, international sample of clinicians to an online research study. *PLoS ONE* **13**, e0200184, doi:10.1371/journal.pone.0200184 (2018).

37 Liu, M. & Wronski, L. Examining completion rates in web surveys via over 25,000 real-world surveys. *Social Science Computer Review* **36**, 116-124 (2018).

38 Bosnjak, M. & Tuten, T. L. Classifying Response Behaviors in Web-based Surveys. *Journal of Computer-Mediated Communication* **6** (2001).

39 OECD & European Union. Health at a Glance: Europe 2020. State of Health in the EU Cycle. OECD Publishing; Paris. Available from: https://doi.org/10.1787/82129230-en [accessed 26 Nov 2021]. (2020).

40 Aiken, L. H., Sloane, D. M., Ball, J. *et al.* Patient satisfaction with hospital care and nurses in England: an observational study. *BMJ Open* **8**, e019189, doi:10.1136/bmjopen-2017-019189 (2018).

41 Saliba, W. & Ostojic, P. Personality and participation: who volunteers to participate in studies. *Psychology* **5**, 230-243, doi:10.4236/psych.2014.53034 (2014).

42 Cousino, M. K., Pasquali, S. K., Romano, J. C. *et al.* Impact of the COVID-19 pandemic on CHD care and emotional wellbeing. *Cardiol Young* **31**, 822-828, doi:https://dx.doi.org/10.1017/S1047951120004758 (2021).

43 Gali, K., Joshi, S., Hueneke, S. *et al.* Barriers, access and management of paediatric epilepsy with telehealth. *J Telemed Telecare* **28**, 213-223, doi:https://dx.doi.org/10.1177/1357633X20969531 (2022).

44 Pooni, R., Pageler, N. M., Sandborg, C. *et al.* Pediatric subspecialty telemedicine use from the patient and provider perspective. *Pediatric Research* **91**, 241-246, doi:https://dx.doi.org/10.1038/s41390-021-01443-4 (2022).

45 European Commission and EUROCAT. Prevalence charts and data on congenital anomalies. Available from: https://eu-rd-platform.jrc.ec.europa.eu/eurocat/eurocat-data/prevalence_en [accessed 30 Nov 2021] (2021).

46 Marino, L. V., Wagland, R., Culliford, D. J. *et al.* "No Official Help Is Available"-Experience of Parents and Children With Congenital Heart Disease During COVID-19. *World Journal for Pediatric and Congenital Heart Surgery* **12**, 500-507, doi:10.1177/21501351211007102 (2021).

47 Darlington, A., Morgan, J., Wagland, R. *et al.* COVID-19 and children with cancer: Parents' experiences, anxieties and support needs. *Pediatr Blood Cancer* **68**, e28790, doi:10.1002/pbc.28790 (2021).

For peer review only - http://bmjopen.bmj.com/site/about/guidelines.xhtml

FIGURE LEGEND

Figure 1 Proportion* of participants reporting 'cancelled or postponed' routine appointments, planned tests or procedures, and planned surgeries with 95% confidence intervals, by country.

*Adjusted by congenital anomaly type, parental age, and education level.

Figure 2 Proportion* of participants reporting that their child's health had been 'moderately to severely' compromised following changes to their child's treatment with 95% confidence intervals, by country.

*Adjusted by congenital anomaly type, parental age, and education level.

Figure 3 Proportion* of participants reporting that their child's physical health was 'worse', 'about the same' or 'better' than it was prior to the pandemic with 95% confidence intervals, by country.

*Adjusted by congenital anomaly type, parental age, and education level.

Figure 4 Proportion* of participants reporting they would have liked more support during the pandemic 'very much' with 95% confidence intervals, by country.

*Adjusted by congenital anomaly type, parental age, and education level.

For peer review only

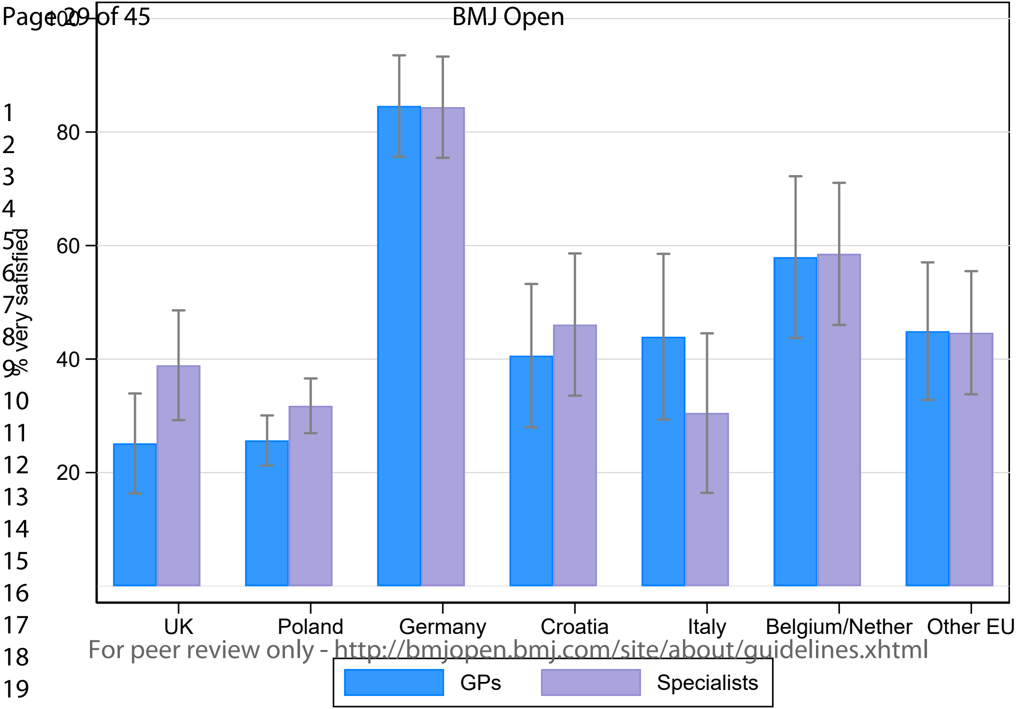

100
80
60
40
20
% very satisfied
UK
Poland
Germany
Croatia
Italy
Belgium/Nether
Other EU
GPs
Specialists

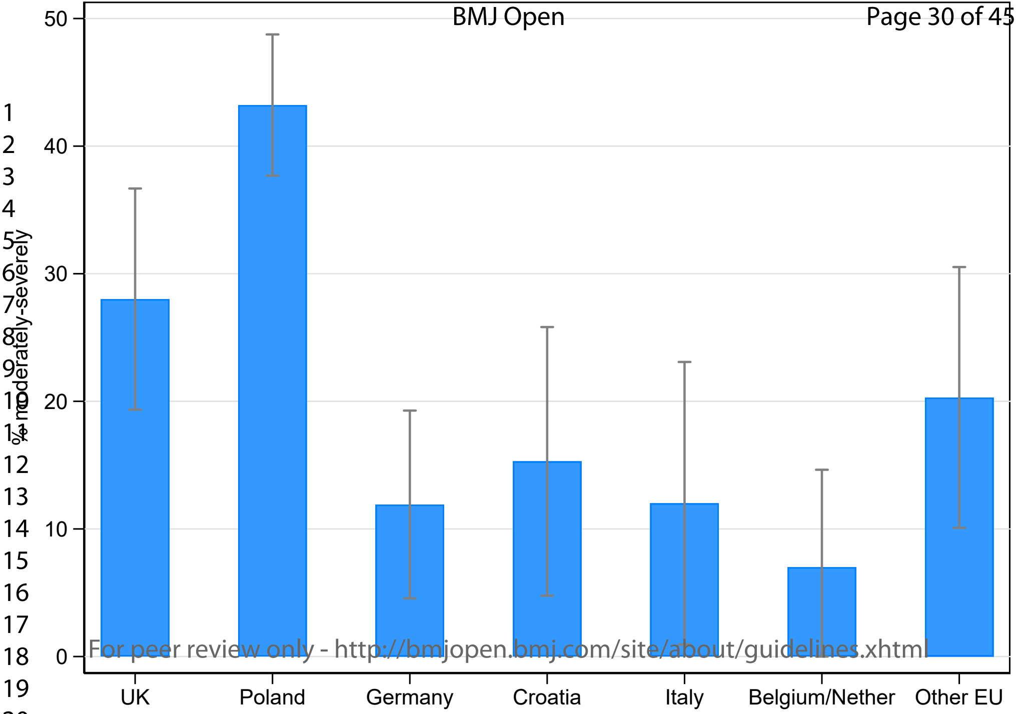

For peer review only - http://bmjopen.bmj.com/site/about/guidelines.xhtml

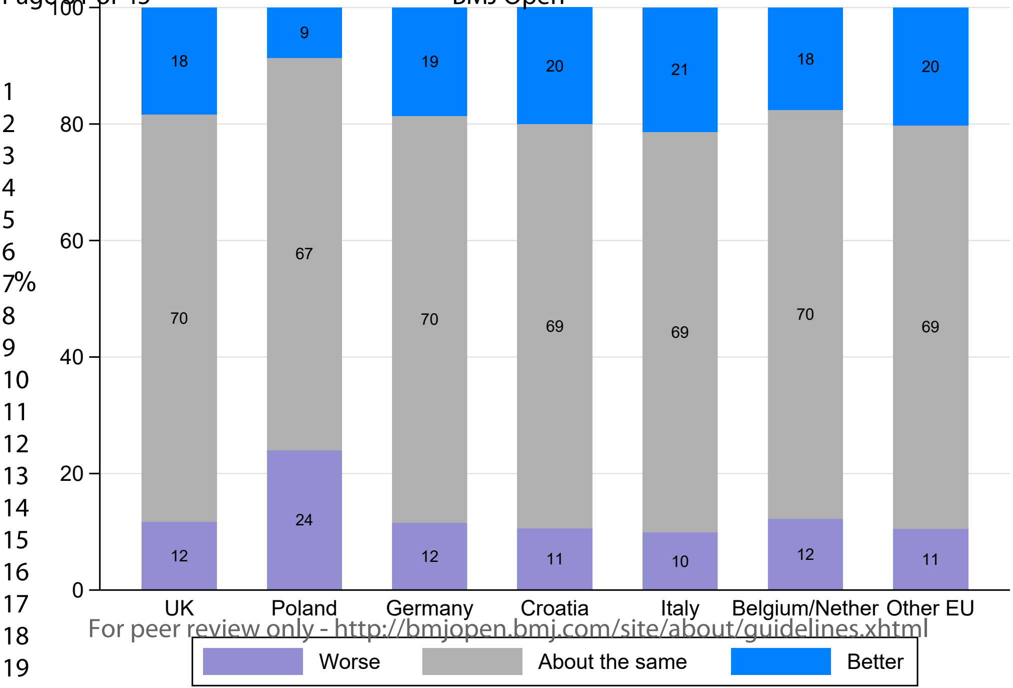

For peer review only - http://bmjopen.bmj.com/site/about/guidelines.xhtml

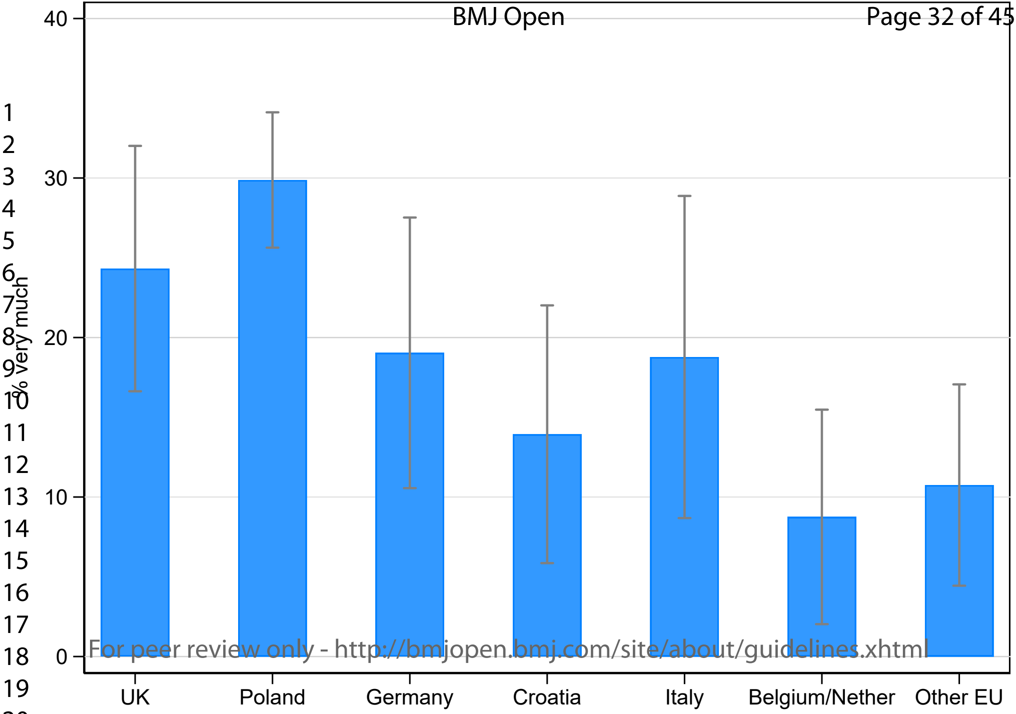

For peer review only - http://bmjopen.bmj.com/site/about/guidelines.xhtml

SUPPLEMENTARY FILE

(A) SURVEY ITEMS

(1) Parent Demographics

1. Which country do you live in? [drop-down list]

2. What type of area do you live in?

a. City (population over 500,000)
b. Large town (population between 100,000 and 500,000)
c. Medium town (population between 20,000 and 100,000)
d. Small town (population less than 20,000)
e. Suburban village
f. Village
g. Rural/isolated area (e.g. a farm)

3. What is your age?

a. Less than 20 years
b. 20-25 years
c. 26-30 years
d. 31-35 years
e. 36-40 years
f. 41-45 years
g. 46-50 years
h. More than 50 years

4. What is the highest level of education you have completed?

a. Primary school
b. Secondary school up to 16 years
c. Secondary or further education after 16 years
d. University
e. Post-graduate / Doctoral studies

5. What is your employment status?

a. Employed (full-time), including self-employed
b. Employed (part-time), including self-employed
c. Full-time homemaker/carer
d. Long-term sick/disabled
e. Retired
f. Student
g. Unemployed
h. On furlough

6. How long have you lived in your country of residence?

a. Up to 1 year
b. Between 1-5 years
c. Between 6-10 years
d. More than 10 years
e. From birth

For peer review only - http://bmjopen.bmj.com/site/about/guidelines.xhtml

f. Prefer not to say

7. What is your relationship to the child this survey is about?

a. Mother (biological)
b. Mother (adoptive)
c. Father (biological)
d. Father (adoptive)
e. Legal guardian related to the child
f. Legal guardian unrelated to the child / foster parent
g. Another family member

8. Is your child being raised with any siblings?

a. Yes, biological sibling(s)
b. Yes, adoptive sibling(s)
c. Yes, biological and adoptive siblings
d. No [survey skips to next section]

9. Is their sibling/are their siblings older or younger than the child this survey is about?

a. Older
b. Younger
c. Both older and younger
d. Same age (twin)

(2) Child Demographics and Medical Information

1. What age is your child?

a. Less than 1 year
b. 1-3 years
c. 4-6 years
d. 7-10 years

2. What is your child's gender?

a. Male
b. Female
c. Other
d. Prefer not to say

3. Which of the following conditions has your child been diagnosed with? (If your child has more than one of these conditions, please select all that apply)

a. Cleft lip (with or without cleft palate)
b. Spina bifida
c. Congenital heart defect that required surgical intervention
d. Down's syndrome

4. Was your child's [*condition*] detected prenatally (during pregnancy)?

a. Yes [survey moves to question 5]
b. No [survey skips to question 6]
c. I don't know [survey skips to question 6]

For peer review only - http://bmjopen.bmj.com/site/about/guidelines.xhtml

5. In which week of pregnancy was your child's [*condition*] detected?

a. Before 13 weeks
b. Between 14 and 21 weeks
c. At 22 weeks or later
d. I'm not sure

6. Does your child have any other congenital anomalies (conditions present from birth)?

a. Yes

Please select all that apply:

- Brain anomalies
- Hydrocephalus
- Eye anomalies
- Anomalies of face, ear and neck
- Lung anomalies
- Abdominal anomalies
- Renal anomalies
- Genital anomalies
- Skeletal anomalies
- Limb anomalies
- Chromosomal or genetic abnormality (other than Down's syndrome)
- Other anomaly

b. No

7. Does your child have any other health conditions?

a. Yes

Please select all that apply:

- Autism or attention disorder
- Learning disability
- Epilepsy
- Cerebral Palsy
- Asthma
- Allergy or food intolerance
- Eczema or other skin disease
- Recurrent infections
- Hearing loss
- Vision problems
- Celiac disease
- Diabetes
- Endocrine disorder
- Immune disorder
- Blood disorder
- Cancer
- Other

b. No

For peer review only

(3) Provision of healthcare

1. Has your child had any routine appointments postponed or cancelled?

   a. Yes
   b. No
   c. Not applicable (they have not had any routine appointments in this period)

2. Has your child had any planned surgeries postponed or cancelled?

   a. Yes
   b. No
   c. Not applicable (they have not had any planned surgeries in this period)

3. Has your child had any planned tests or procedure's postponed or cancelled?

   a. Yes
   b. No
   c. Not applicable (they have not had any planned tests or procedures in this period)

4. Has your child had any face-to-face appointments re-scheduled as virtual appointments (e.g. by telephone or online)?

   a. Yes
   b. No
   c. Not applicable (they have not had any appointments in this period)

5. [If yes] Overall, how do you rate the quality of your virtual appointments?

   a. Poor
   b. Fair
   c. Good
   d. Very good

7. Have you had any difficulty accessing medication for your child?

   a. Not at all
   b. A little
   c. Quite a bit
   d. Very much
   e. Not applicable (they do not require medication)

(4) Impact on the child

1. Has your child's health been compromised by any changes to ongoing treatment for their condition (e.g. medication, physical therapy, or surgery)?

   a. Not at all
   b. Slightly
   c. Moderately
   d. Severely
   e. Not applicable (there have been no changes to their ongoing treatment)
   f. Not applicable (they have not had any ongoing treatment)

For peer review only - http://bmjopen.bmj.com/site/about/guidelines.xhtml

For peer review only

2. Compared to before COVID-19, how would you rate your child's physical health?

a. Much worse
b. Somewhat worse
c. About the same
d. Somewhat better
e. Much better

3. Compared to before COVID-19, how would you rate your child's emotional well-being?

a. Much worse
b. Somewhat worse
c. About the same
d. Somewhat better
e. Much better

(5) Support for Parents

1. During the pandemic, to what extent have you felt satisfied with the support you have received from the following people/organisations? (If you have not needed or requested support from a listed source please select N/A)

	Not at all satisfied	Slightly satisfied	Moderately satisfied	Very satisfied	N/A
General practitioner					
Specialist doctor or specialist nurse					
Partner (or person I am closest to)					
Friends and family					
Parents of children with the same health condition					
Patient/parent organisation					
School					

2. Overall, would you have liked more support during the COVID-19 pandemic?

a. Not at all
b. A little
c. Quite a bit
d. Very much

For peer review only

(B) STROBE CHECKLIST

STROBE Statement—Checklist of items that should be included in reports of ***cross-sectional studies***

	Item No	Recommendation	Page No
**Title and abstract**	1	(*a*) Indicate the study's design with a commonly used term in the title or the abstract	1, 3
		(*b*) Provide in the abstract an informative and balanced summary of what was done and what was found	3
**Introduction**			
Background/rationale	2	Explain the scientific background and rationale for the investigation being reported	4-5
Objectives	3	State specific objectives, including any prespecified hypotheses	5
**Methods**			
Study design	4	Present key elements of study design early in the paper	5, 7
Setting	5	Describe the setting, locations, and relevant dates, including periods of recruitment, exposure, follow-up, and data collection	5-7
Participants	6	(*a*) Give the eligibility criteria, and the sources and methods of selection of participants	7
Variables	7	Clearly define all outcomes, exposures, predictors, potential confounders, and effect modifiers. Give diagnostic criteria, if applicable	7-8
Data sources/ measurement	8*	For each variable of interest, give sources of data and details of methods of assessment (measurement). Describe comparability of assessment methods if there is more than one group	7
Bias	9	Describe any efforts to address potential sources of bias	n/a
Study size	10	Explain how the study size was arrived at	9
Quantitative variables	11	Explain how quantitative variables were handled in the analyses. If applicable, describe which groupings were chosen and why	9
Statistical methods	12	(*a*) Describe all statistical methods, including those used to control for confounding	9-10
		(*b*) Describe any methods used to examine subgroups and interactions	9-10
		(*c*) Explain how missing data were addressed	9-10
		(*d*) If applicable, describe analytical methods taking account of sampling strategy	n/a
		(*e*) Describe any sensitivity analyses	n/a

For peer review only - http://bmjopen.bmj.com/site/about/guidelines.xhtml

**Results**			
Participants	13*	(a) Report numbers of individuals at each stage of study—eg numbers potentially eligible, examined for eligibility, confirmed eligible, included in the study, completing follow-up, and analysed	10
		(b) Give reasons for non-participation at each stage	n/a
		(c) Consider use of a flow diagram	n/a
Descriptive data	14*	(a) Give characteristics of study participants (eg demographic, clinical, social) and information on exposures and potential confounders	6, 10
		(b) Indicate number of participants with missing data for each variable of interest	10 (and Tables 2-5)
Outcome data	15*	Report numbers of outcome events or summary measures	n/a
Main results	16	(*a*) Give unadjusted estimates and, if applicable, confounder-adjusted estimates and their precision (eg, 95% confidence interval). Make clear which confounders were adjusted for and why they were included	11-14, 16 (Tables 2-5)
		(*b*) Report category boundaries when continuous variables were categorized	n/a
		(*c*) If relevant, consider translating estimates of relative risk into absolute risk for a meaningful time period	n/a
Other analyses	17	Report other analyses done—eg analyses of subgroups and interactions, and sensitivity analyses	n/a
**Discussion**			
Key results	18	Summarise key results with reference to study objectives	17
Limitations	19	Discuss limitations of the study, taking into account sources of potential bias or imprecision. Discuss both direction and magnitude of any potential bias	18-19
Interpretation	20	Give a cautious overall interpretation of results considering objectives, limitations, multiplicity of analyses, results from similar studies, and other relevant evidence	17-18
Generalisability	21	Discuss the generalisability (external validity) of the study results	18
**Other information**			
Funding	22	Give the source of funding and the role of the funders for the present study and, if applicable, for the original study on which the present article is based	21-22

*Give information separately for exposed and unexposed groups. **Note:** An Explanation and Elaboration article discusses each checklist item and gives methodological background and published examples of transparent reporting. The STROBE checklist is best used in conjunction with this article (freely available on the Web sites of PLoS Medicine at http://www.plosmedicine.org/, Annals of Internal Medicine at http://www.annals.org/, and Epidemiology at http://www.epidem.com/). Information on the STROBE Initiative is available at www.strobe-statement.org.

For peer review only - http://bmjopen.bmj.com/site/about/guidelines.xhtml

(C) LOGISTIC REGRESSION FINDINGS

Table 1 Logistic regression model output for cancelled/postponed routine appointments

**Routine Appointments**	Odds ratio	Standard error	z	P>|z|	[95% conf. interval]	
**Country**						
UK	1.70	0.52	1.73	0.084	0.93	3.11
Germany	0.11	0.03	-7.8	0.000	0.06	0.19
Croatia	0.20	0.06	-5.62	0.000	0.11	0.35
Italy	0.62	0.20	-1.46	0.144	0.32	1.18
Belgium/Nether	0.16	0.05	-6.37	0.000	0.09	0.28
Other	0.34	0.09	-4.21	0.000	0.20	0.56
**Age**	1.32	0.17	2.19	0.029	1.03	1.69
**Education**	1.14	0.15	0.99	0.325	0.88	1.46
**CA type**						
Cleft Lip	1.03	0.22	0.15	0.883	0.68	1.55
Spina bifida	1.47	0.39	1.46	0.143	0.88	2.48
Down syndrome	1.85	0.41	2.78	0.005	1.20	2.86
DS + CHD	2.09	0.82	1.88	0.061	0.97	4.53
_cons	1.36	0.47	0.89	0.373	0.69	2.67

CHD – congenital heart defect; CA – congenital anomaly.
Comparator groups were Poland for country, and CHD for CA type

For peer review only

Table 2 Logistic regression model output for cancelled/postponed surgeries

**Surgeries**	Odds ratio	Standard error	z	P>|z|	[95% conf. interval]	
**Country**						
UK	0.91	0.27	-0.3	0.761	0.51	1.65
Germany	0.16	0.08	-3.68	0.000	0.06	0.43
Croatia	0.27	0.14	-2.6	0.009	0.10	0.73
Italy	0.21	0.12	-2.83	0.005	0.07	0.62
Belgium/Nether	0.34	0.14	-2.71	0.007	0.16	0.74
Other	0.25	0.10	-3.41	0.001	0.11	0.56
**Age**	0.83	0.14	-1.12	0.263	0.61	1.15
**Education**	1.33	0.20	1.83	0.068	0.98	1.79
**CA type**						
Cleft Lip	1.79	0.46	2.29	0.022	1.09	2.96
Spina bifida	1.19	0.42	0.49	0.622	0.60	2.38
Down syndrome	0.97	0.29	-0.09	0.927	0.54	1.75
DS + CHD	1.05	0.49	0.1	0.918	0.42	2.63
_cons	0.40	0.18	-2.06	0.04	0.17	0.96

CHD – congenital heart defect; CA – congenital anomaly.
Comparator groups were Poland for country, and CHD for CA type

Table 3 Logistic regression model output for cancelled/postponed test and procedures

**Tests/procedures**	Odds ratio	Standard error	z	P>|z|	[95% conf. interval]	
**Country**						
UK	1.08	0.27	0.3	0.767	0.66	1.74
Germany	0.11	0.04	-6.55	0.000	0.06	0.21
Croatia	0.29	0.09	-3.9	0.000	0.15	0.54
Italy	0.61	0.20	-1.51	0.132	0.33	1.16
Belgium/Nether	0.15	0.05	-5.31	0.000	0.07	0.30
Other	0.39	0.10	-3.69	0.000	0.23	0.64
**Age**	1.18	0.15	1.32	0.185	0.92	1.52
**Education**	1.15	0.14	1.16	0.246	0.91	1.47
**CA type**						
Cleft Lip	1.45	0.31	1.75	0.08	0.96	2.21
Spina bifida	2.78	0.74	3.86	0.000	1.65	4.67
Down syndrome	2.40	0.52	4.05	0.000	1.57	3.66
DS + CHD	1.69	0.60	1.47	0.142	0.84	3.41
_cons	0.65	0.23	-1.23	0.218	0.33	1.29

CHD – congenital heart defect; CA – congenital anomaly.
Comparator groups were Poland for country, and CHD for CA type

For peer review only

For peer review only - http://bmjopen.bmj.com/site/about/guidelines.xhtml

Table 4 Logistic regression model output for problems accessing medication (a little-very)

**Accessing medication**	Odds ratio	Standard error	z	P>|z|	[95% conf. interval]	
**Country**						
UK	1.42	0.36	1.39	0.165	0.87	2.32
Germany	0.14	0.07	-3.97	0.000	0.06	0.38
Croatia	0.08	0.06	-3.37	0.001	0.02	0.35
Italy	0.30	0.15	-2.38	0.017	0.11	0.81
Belgium/Nether	0.43	0.17	-2.17	0.030	0.20	0.92
Other	0.16	0.07	-4.07	0.000	0.07	0.39
**Age**	0.91	0.13	-0.65	0.518	0.69	1.21
**Education**	0.87	0.13	-0.95	0.340	0.65	1.16
**CA type**						
Cleft Lip	0.35	0.10	-3.66	0.000	0.20	0.61
Spina bifida	0.71	0.20	-1.23	0.218	0.41	1.23
Down syndrome	0.58	0.14	-2.23	0.026	0.36	0.94
DS + CHD	0.36	0.16	-2.37	0.018	0.15	0.84
_cons	1.24	0.49	0.56	0.578	0.58	2.68

CHD – congenital heart defect; CA – congenital anomaly.
Comparator groups were Poland for country, and CHD for CA type

Table 5 Logistic regression model output for face-to-face appointments re-scheduled as virtual

**Face-to-face rescheduled**	Odds ratio	Standard error	z	P>|z|	[95% conf. interval]	
**Country**						
UK	2.84	0.88	3.38	0.001	1.55	5.20
Germany	0.12	0.04	-7.17	0.000	0.07	0.21
Croatia	0.31	0.10	-3.76	0.000	0.17	0.57
Italy	0.21	0.07	-4.64	0.000	0.11	0.40
Belgium/Nether	0.18	0.06	-5.34	0.000	0.10	0.34
Other	0.41	0.10	-3.53	0.000	0.25	0.67
**Age**	1.15	0.15	1.07	0.284	0.89	1.48
**Education**	1.06	0.14	0.49	0.626	0.83	1.36
**CA type**						
Cleft Lip	1.37	0.29	1.51	0.131	0.91	2.07
Spina bifida	2.82	0.75	3.93	0.000	1.68	4.74
Down syndrome	3.29	0.71	5.47	0.000	2.14	5.03
DS + CHD	5.39	2.31	3.94	0.000	2.33	12.46
_cons	0.95	0.33	-0.16	0.873	0.48	1.88

CHD – congenital heart defect; CA – congenital anomaly.
Comparator groups were Poland for country, and CHD for CA type

For peer review only - http://bmjopen.bmj.com/site/about/guidelines.xhtml

Table 6 Logistic regression model output for the quality of virtual appointments (poor)

Quality of virtual appointments (poor)	Odds ratio	Standard error	z	P>|z|	[95% conf. interval]	
**Country**						
UK	0.44	0.12	-2.89	0.004	0.26	0.77
Germany	1.00†	(empty)				
Croatia	0.32	0.18	-1.99	0.047	0.11	0.98
Italy	0.62	0.35	-0.84	0.403	0.21	1.89
Belgium/Nether	0.09	0.09	-2.35	0.019	0.01	0.67
Other	0.46	0.18	-1.98	0.048	0.22	0.99
**Age**	0.95	0.15	-0.35	0.724	0.70	1.28
**Education**	0.56	0.09	-3.59	0	0.41	0.77
**CA type**						
Cleft Lip	0.72	0.21	-1.11	0.267	0.40	1.28
Spina bifida	0.65	0.22	-1.29	0.196	0.33	1.25
Down syndrome	0.71	0.19	-1.3	0.192	0.42	1.19
DS + CHD	0.65	0.29	-0.97	0.333	0.28	1.54
_cons	2.44	1.08	2.01	0.044	1.02	5.82

†0 participants in Germany rated virtual appointments as poor
CHD – congenital heart defect; CA – congenital anomaly. Comparator groups were Poland for country, and CHD for CA type

Table 6 Logistic regression model output for satisfaction with support from general practitioner

Support from GP	Odds ratio	Standard error	z	P>|z|	[95% conf. interval]	
**Country**						
UK	0.97	0.26	-0.1	0.920	0.57	1.65
Germany	16.28	6.08	7.47	0.000	7.83	33.86
Croatia	1.99	0.59	2.32	0.020	1.11	3.56
Italy	2.29	0.76	2.5	0.013	1.19	4.38
Belgium/Nether	4.05	1.33	4.27	0.000	2.13	7.69
Other	2.38	0.67	3.09	0.002	1.37	4.13
**Age**	0.90	0.12	-0.85	0.395	0.69	1.16
**Education**	0.95	0.12	-0.43	0.669	0.73	1.22
**CA type**						
Cleft Lip	0.76	0.18	-1.15	0.251	0.48	1.21
Spina bifida	1.38	0.38	1.16	0.247	0.80	2.36
Down syndrome	1.14	0.25	0.61	0.540	0.74	1.76
DS + CHD	1.45	0.56	0.97	0.333	0.68	3.07
_cons	0.46	0.17	-2.16	0.031	0.23	0.93

CHD – congenital heart defect; CA – congenital anomaly.
Comparator groups were Poland for country, and CHD for CA type

For peer review only

For peer review only - http://bmjopen.bmj.com/site/about/guidelines.xhtml

Table 7 Logistic regression model output for satisfaction with support from specialist doctor/nurse

Support from Specialist	Odds ratio	Standard error	z	P>|z|	[95% conf. interval]	
**Country**						
UK	1.38	0.33	1.32	0.188	0.86	2.21
Germany	12.10	4.46	6.76	0.000	5.87	24.92
Croatia	1.86	0.53	2.14	0.032	1.05	3.26
Italy	0.94	0.34	-0.17	0.868	0.46	1.92
Belgium/Nether	3.09	0.90	3.87	0.000	1.75	5.48
Other	1.75	0.45	2.18	0.029	1.06	2.89
**Age**	1.00	0.13	0.03	0.972	0.79	1.28
**Education**	1.04	0.13	0.34	0.735	0.82	1.34
**CA type**						
Cleft Lip	1.10	0.24	0.43	0.664	0.72	1.67
Spina bifida	0.94	0.25	-0.22	0.825	0.57	1.57
Down syndrome	0.55	0.12	-2.8	0.005	0.36	0.83
DS + CHD	1.06	0.39	0.16	0.876	0.51	2.18
_cons	0.49	0.17	-2.08	0.038	0.25	0.96

CHD – congenital heart defect; CA – congenital anomaly.
Comparator groups were Poland for country, and CHD for CA type

Table 8 Logistic regression model output for satisfaction with support from schools

Support from schools	Odds ratio	Standard error	z	P>|z|	[95% conf. interval]	
**Country**						
UK	2.40	0.80	2.63	0.009	1.25	4.61
Germany	1.76	0.90	1.11	0.267	0.65	4.81
Croatia	0.63	0.37	-0.79	0.431	0.19	2.01
Italy	1.96	0.74	1.78	0.076	0.93	4.11
Belgium/Nether	1.54	0.62	1.07	0.287	0.70	3.41
Other	1.32	0.49	0.75	0.455	0.64	2.75
**Age**	0.96	0.19	-0.23	0.822	0.65	1.41
**Education**	0.92	0.16	-0.46	0.645	0.66	1.30
**CA type**						
Cleft Lip	1.02	0.35	0.05	0.959	0.52	1.99
Spina bifida	1.37	0.50	0.85	0.393	0.67	2.81
Down syndrome	1.64	0.51	1.62	0.106	0.90	3.00
DS + CHD	1.53	0.89	0.74	0.459	0.49	4.76
_cons	0.37	0.21	-1.76	0.078	0.12	1.12

CHD – congenital heart defect; CA – congenital anomaly.
Comparator groups were Poland for country, and CHD for CA type

For peer review only - http://bmjopen.bmj.com/site/about/guidelines.xhtml

Table 9 Logistic regression model output for satisfaction with support from partner

**Support from partner**	Odds ratio	Standard error	z	P>|z|	[95% conf. interval]	
**Country**						
UK	1.62	0.42	1.87	0.062	0.98	2.70
Germany	3.61	1.62	2.86	0.004	1.50	8.70
Croatia	1.79	0.58	1.79	0.073	0.95	3.38
Italy	1.09	0.37	0.25	0.804	0.56	2.13
Belgium/Nether	0.84	0.24	-0.64	0.523	0.48	1.45
Other	1.01	0.27	0.02	0.981	0.60	1.69
**Age**	1.04	0.13	0.29	0.770	0.81	1.32
**Education**	1.01	0.13	0.06	0.954	0.79	1.28
**CA type**						
Cleft Lip	1.63	0.36	2.22	0.026	1.06	2.50
Spina bifida	1.70	0.48	1.88	0.061	0.98	2.97
Down syndrome	1.11	0.23	0.51	0.607	0.74	1.66
DS + CHD	1.15	0.41	0.38	0.702	0.57	2.32
_cons	1.71	0.59	1.53	0.125	0.86	3.37

CHD – congenital heart defect; CA – congenital anomaly.
Comparator groups were Poland for country, and CHD for CA type

Table 10 Logistic regression model output for satisfaction with support from friends and family

**Support from friends/family**	Odds ratio	Standard error	z	P>|z|	[95% conf. interval]	
**Country**						
UK	0.62	0.13	-2.22	0.027	0.40	0.95
Germany	1.77	0.55	1.81	0.070	0.96	3.27
Croatia	1.13	0.32	0.44	0.659	0.65	1.95
Italy	0.56	0.17	-1.89	0.059	0.30	1.02
Belgium/Nether	0.61	0.16	-1.86	0.062	0.36	1.03
Other	0.71	0.17	-1.44	0.151	0.44	1.14
**Age**	0.89	0.10	-1.08	0.280	0.71	1.10
**Education**	1.12	0.13	1.03	0.303	0.90	1.40
**CA type**						
Cleft Lip	1.15	0.23	0.71	0.478	0.78	1.69
Spina bifida	0.92	0.22	-0.34	0.731	0.57	1.48
Down syndrome	1.11	0.21	0.54	0.586	0.77	1.60
DS + CHD	1.17	0.38	0.47	0.636	0.62	2.21
_cons	1.56	0.48	1.43	0.151	0.85	2.86

CHD – congenital heart defect; CA – congenital anomaly.
Comparator groups were Poland for country, and CHD for CA type

For peer review only

Table 11 Logistic regression model output for satisfaction with support from other parents

Support from other parents	Odds ratio	Standard error	z	P>|z|	[95% conf. interval]	
**Country**						
UK	0.52	0.13	-2.6	0.009	0.32	0.85
Germany	0.77	0.32	-0.64	0.520	0.34	1.73
Croatia	0.89	0.27	-0.4	0.693	0.49	1.60
Italy	0.39	0.15	-2.47	0.013	0.18	0.82
Belgium/Nether	0.25	0.10	-3.45	0.001	0.11	0.55
Other	0.64	0.19	-1.54	0.122	0.36	1.13
**Age**	0.94	0.13	-0.47	0.636	0.71	1.23
**Education**	1.06	0.14	0.46	0.647	0.82	1.39
**CA type**						
Cleft Lip	1.60	0.39	1.93	0.054	0.99	2.57
Spina bifida	0.73	0.22	-1.03	0.303	0.41	1.32
Down syndrome	1.07	0.24	0.32	0.749	0.70	1.65
DS + CHD	1.25	0.49	0.57	0.572	0.58	2.68
_cons	1.84	0.69	1.64	0.101	0.89	3.82

CHD – congenital heart defect; CA – congenital anomaly.
Comparator groups were Poland for country, and CHD for CA type

Table 12 Logistic regression model output for satisfaction with support from patient organisations

Support from patient organisations	Odds ratio	Standard error	z	P>|z|	[95% conf. interval]	
**Country**						
UK	0.48	0.13	-2.76	0.006	0.28	0.81
Germany	1.14	0.52	0.28	0.780	0.46	2.81
Croatia	0.45	0.16	-2.21	0.027	0.22	0.91
Italy	0.39	0.15	-2.4	0.016	0.18	0.84
Belgium/Nether	0.12	0.08	-3.34	0.001	0.03	0.41
Other	0.49	0.15	-2.34	0.019	0.27	0.89
**Age**	0.96	0.15	-0.24	0.808	0.71	1.31
**Education**	0.99	0.15	-0.09	0.931	0.74	1.32
**CA type**						
Cleft Lip	1.46	0.40	1.4	0.162	0.86	2.50
Spina bifida	0.70	0.23	-1.07	0.284	0.36	1.34
Down syndrome	1.03	0.25	0.13	0.899	0.64	1.66
DS + CHD	1.26	0.51	0.57	0.572	0.57	2.81
_cons	1.33	0.55	0.69	0.488	0.59	2.99

CHD – congenital heart defect; CA – congenital anomaly.
Comparator groups were Poland for country, and CHD for CA type

For peer review only

For peer review only - http://bmjopen.bmj.com/site/about/guidelines.xhtml

Table 13 Logistic regression model output for 'overall need for more support'

**Overall more support needed**	Odds ratio	Standard error	z	P>|z|	[95% conf. interval]	
**Country**						
UK	0.75	0.18	-1.18	0.236	0.47	1.21
Germany	0.55	0.17	-1.98	0.048	0.30	0.99
Croatia	0.38	0.14	-2.69	0.007	0.18	0.77
Italy	0.54	0.19	-1.72	0.086	0.27	1.09
Belgium/Nether	0.22	0.10	-3.38	0.001	0.09	0.53
Other	0.28	0.10	-3.59	0.000	0.14	0.56
**Age**	1.02	0.13	0.13	0.898	0.79	1.30
**Education**	0.92	0.11	-0.64	0.520	0.72	1.18
**CA type**						
Cleft Lip	0.53	0.13	-2.68	0.007	0.34	0.85
Spina bifida	0.86	0.23	-0.57	0.569	0.50	1.46
Down syndrome	1.03	0.21	0.14	0.889	0.69	1.54
DS + CHD	1.04	0.36	0.11	0.914	0.53	2.04
_cons	0.54	0.19	-1.76	0.078	0.28	1.07

CHD – congenital heart defect; CA – congenital anomaly.
Comparator groups were Poland for country, and CHD for CA type

For peer review only
